# Literacy Training of Kindergarten Children With Pencil, Keyboard or Tablet Stylus: The Influence of the Writing Tool on Reading and Writing Performance at the Letter and Word Level

**DOI:** 10.3389/fpsyg.2019.03054

**Published:** 2020-01-22

**Authors:** Carmen Mayer, Stefanie Wallner, Nora Budde-Spengler, Sabrina Braunert, Petra A. Arndt, Markus Kiefer

**Affiliations:** ^1^Transfer Centre for Neuroscience and Education, Ulm University, Ulm, Germany; ^2^Section for Cognitive Electrophysiology, Department of Psychiatry, Ulm University, Ulm, Germany; ^3^Department of German Studies, Catholic University of Eichstätt-Ingolstadt, Eichstätt, Germany

**Keywords:** written language acquisition, literacy training, embodied cognition, digital media, preschool children, pencil, tablet, keyboard

## Abstract

During the last years, digital writing devices are increasingly replacing handwriting with pencil and paper. As reading and writing skills are central for education, it is important to know, which writing tool is optimal for initial literacy education. The present training study was therefore set up to test the influence of the writing tool on the acquisition of literacy skills at the letter and word level with various tests in a large sample of kindergarten children (*n* = 147). Using closely matched letter learning games, children were trained with 16 letters by handwriting with a pencil on a sheet of paper, by writing with a stylus on a tablet computer, or by typing letters using a virtual keyboard on a tablet across 7 weeks. Training using a stylus on a touchscreen is an interesting comparison condition for traditional handwriting, because the slippery surface of a touchscreen has lower friction than paper and thus increases difficulty of motor control. Before training, immediately after training and four to five weeks after training, we assessed reading and writing performance using standardized tests. We also assessed visuo-spatial skills before and after training, in order to test, whether the different training regimens affected cognitive domains other than written language. Children of the pencil group showed superior performance in letter recognition and improved visuo-spatial skills compared with keyboard training. The performance of the stylus group did not differ significantly neither from the keyboard nor from the pencil group. Keyboard training, however, resulted in superior performance in word writing and reading compared with handwriting training with a stylus on the tablet, but not compared with the pencil group. Our results suggest that handwriting with pencil fosters acquisition of letter knowledge and improves visuo-spatial skills compared with keyboarding. At least given the current technological state, writing with a stylus on a touchscreen seems to be the least favorable writing tool, possibly because of increased demands on motor control. Future training studies covering a more extended observation period over years are needed to allow conclusions about long-term effects of writing tools on literacy acquisition as well as on general cognitive development.

## Introduction

Since its early beginnings about 5000 years ago in Mesopotamia and Egypt, different tools were used to form symbols aimed to store language for a long period of time in written form: Humans imprinted symbols in clay using a stylus or painted them on papyrus, parchment or paper using reed, feather or pen. These tools used for writing by hand have been complemented by other technologies such as stamps, letterpress or typewriter. Nevertheless, for several 1000 years of past literacy culture, handwriting was an important, if not the prevailing mode for writing (Kiefer and Velay, 2016).

During the last years, however, digital writing devices associated with the use of computers, tablet computers or mobile phones are increasingly replacing handwriting (for overviews, see Mangen and Velay, 2010; Radesky et al., 2015; Kiefer and Velay, 2016). The use of digital devices for writing has impact on basic sensory-motor skills: In adults, a high frequency of keyboard use in written text production in everyday life was related to a decrement of the skill to produce precisely controlled arm–hand movements compared with a high frequency of handwriting (Sulzenbrück et al., 2010; Sulzenbrück et al., 2011; Heuer, 2016). In a recent survey among German teachers, poor sensory-motor skills, which are essential for handwriting, were reported to be deficient in young children entering elementary schools, possibly due to lack of prior training and the use of digital media (Marquardt et al., 2016). In the present days, children may get the first everyday writing experiences by typing on a computer or mobile phone, much before they master handwriting (Mangen and Velay, 2010). Additionally, digital devices such as tablet computers are increasingly introduced to kindergarten and elementary schools for educational purposes (Herzig and Grafe, 2006). Even small children intuitively interact with digital devices by typing or touching (Couse and Chen, 2010; Buchegger, 2013). As reading and writing skills are highly relevant for success at school and in professional life (Gut et al., 2012), it is important to know, which writing tool, handwriting with pen(cil) and paper, handwriting with a stylus on a tablet or typing on a keyboard, is optimal for initial literacy education in school children.

Besides of media education or general instructional purposes, some elementary schools started to implement initial literacy training based on typewriting using digital devices (Spitzer, 2015; Arndt, 2016), whereas handwriting using pencil and paper acquisition is delayed to later grades. Some of these programs are motivated by idea that children’s sensory-motor skills are frequently poor at the entry to elementary school (Genlott and Grönlund, 2013). Learning to write by hand therefore imposes high demands on cognitive control resources in children to acquire the complex motor programs associated with handwriting. These consumed cognitive resources cannot be allocated to other cognitive processes necessary for written language production. In contrast, the motor programs for typing on digital devices are much easier to acquire. It has therefore been proposed, that writing training with typing may accelerate writing in young children, disadvantaged children, or in children with less developed sensory-motor skills (Calhoun, 1985; Castles et al., 2013; Zheng et al., 2013). In particular, it has been claimed that in children with still developing sensory-motor skills more cognitive resources can be allocated for the content of written text production during keyboarding compared with handwriting (Genlott and Grönlund, 2013). Such a view based on the easiness of motor programs associated with writing would predict better reading and writing performance when writing letters and words is trained by typing on a digital device (Genlott and Grönlund, 2013, 2016). In support of this view, a small, but positive correlation between frequency of computer use and letter knowledge has been found in a large cohort of 4-year old children (*n* = 1,539) (Castles et al., 2013). Since tablet computers were not yet common at that time, the result indicates that using the keyboard appears to support the acquisition of letter knowledge in young children.

Several studies investigated the effects of computer usage on disadvantaged children at various ages. Mentally retarded teenage males (aged 12.7–14.11) developed faster and more accurate writing after typing than after handwriting training (Calhoun, 1985). In a sample of 2,158 upper elementary students, it was shown that especially at-risk students were able to achieve significant gains in writing when participating in a laptop program (Zheng et al., 2013).

Genlott and Grönlund (2013) investigated the effects of a literacy training program based on keyboarding compared to traditional handwriting training with a sample of *n* = 87 1st grade students (age: 7 years). A group of 41 students received the integrated Write-to-Read (iWTR) keyboarding literacy training. The program includes rich interactions with peers and teachers: Students produce texts on the keyboard, share it with classmates and teachers, receive feedback, discuss and develop their texts. In the control group (*n* = 46 students) teaching was “done as usual” with pencil and paper without further specifications. The application of digital devices within iWTR led to better reading and writing performance compared to the control group. In a second study the iWTR program was extended to WTL (Write to Learn): Mathematics was added to the program and the method was used in all school subjects where abilities as reading, writing, collaboration, and reasoning are important (Genlott and Grönlund, 2016). A sample of 247 students learned with the WTL program. The group was compared to a sample of 128 students who received traditional teaching without systematic use of digital devices, and with a second control group of 127 students who used digital devices during the learning process continuously but individually (i.e., without structured digital feedback and cooperation). Students attended their respective program from 1st to 3rd grade. Testing at the 3rd grade revealed higher literacy as well as mathematics scores in the WTL group compared to either control group. Individual training based on keyboarding without a social component yielded the same literacy performance as traditional training (but poorest performance in mathematics). Although these two studies seem to favor keyboarding over handwriting, they show that interactions with peers might be the essential factor for improved literacy performance in the iWRT and WTL programs, but not the use of a digital device *per se*. Due to testing in grade 3 the outcomes reported in the latter study reflect not only initial literacy training (and mathematics training), but long-term effects of language education.

However, when comparing handwriting with typing, not only the easiness of the motor programs, but also their potential to support letter and word knowledge must be considered (Kiefer and Velay, 2016). In this respect, handwriting and typing have fundamentally different properties (Mangen and Velay, 2010; Mangen and Balsvik, 2016): Handwriting requires carefully reproducing the shape of each letter, whereas in typewriting the motor program is not related to the letter shape and, as a result, no such grapho-motor component is present. Hence, motor programs associated with handwriting provide an additional informative memory trace and may contribute to the representation of the shape of a letter (James and Engelhardt, 2012; Vinci-Booher et al., 2016).

Such interactions between action and perception are important elements of embodied or grounded cognition theories. This type of theories states that cognition is essentially grounded in modality-specific sensory and motor systems (Lakoff and Johnson, 1999; Barsalou et al., 2003; Gallese and Lakoff, 2005; Pulvermüller, 2005; Kiefer and Pulvermüller, 2012; Kiefer and Velay, 2016; Mangen and Balsvik, 2016). Depending on the specific sensory-motor experience, learning establishes modality-specific memory traces, which are partially reactivated during retrieval. In support of the grounded cognition view, action representations have been shown to facilitate recognition of objects with similar action affordances (Helbig et al., 2006; Kiefer et al., 2011; Sim et al., 2014). Furthermore, when participants have to acquire the names and the meaning of novel objects, performing a meaningful action toward an object during training facilitates learning compared with a meaningless pointing action (Kiefer et al., 2007; Soden-Fraunhofen et al., 2008). These results suggest that sensory-motor experiences during training must be meaningfully related to the learning target to result in stronger sensory-motor memory traces that facilitate recognition performance (Kiefer and Trumpp, 2012).

Similar mechanisms of action-perception coupling may also influence letter recognition, reading and writing performance. In line with this suggestion, several training studies in preschool children and adults showed that handwriting training of new letters not only improved spelling accuracy (Cunningham and Stanovich, 1990), but also resulted in superior letter recognition in a subsequent test compared with typing training (Naka, 1998; Longcamp et al., 2005; Longcamp et al., 2008). This suggests that handwriting, which links rich sensory-motor representations to perceptual letter shapes, fosters reading performance compared with typewriting. This link between letter shape and motor programs established through handwriting is also demonstrated by neuroimaging studies: Visual recognition of familiar letters activated not only visual brain areas, but also motor areas (Longcamp et al., 2003; James and Gauthier, 2006; Longcamp et al., 2011). When novel letters were trained by handwriting, an activation in motor areas was observed, which was absent when these novel letters were trained by typing (James and Atwood, 2008; Longcamp et al., 2008). Furthermore, handwriting experience also seems to be necessary in children to develop the adult-like neuronal circuit of letter processing (James and Engelhardt, 2012) by increasing functionally connectivity between visual and motor brain regions (Vinci-Booher et al., 2016).

Although several behavioral and neuroimaging intervention studies seem to suggest a superiority of handwriting training over typing training on subsequent reading and writing performance in young children, results are mixed. Improved letter recognition after handwriting training compared typing training was not always replicated (Kiefer et al., 2015). For instance, in an earlier training study in kindergarten children from our group, letter recognition performance was comparable after handwriting and typewriting training (Kiefer et al., 2015). This absence of training regimen differences for letter recognition in this previous work, however, might be due to a high performance level before training, thereby attenuating differential training effects. In the more difficult word writing and word reading tasks, superior performance after handwriting training was obtained, albeit for word reading this effect was not significant (Kiefer et al., 2015). Unfortunately, effects at the word level are also heterogeneous: A superiority of handwriting over typing training on word writing performance (Cunningham and Stanovich, 1990; Kiefer et al., 2015) was not found in other studies (Vaughn et al., 1992; Ouellette and Tims, 2014). The mixed results may arise from the relatively short training programs, heterogeneity of the samples with regard to age and prior letter knowledge and from different training and test procedures (Kiefer et al., 2015). Furthermore, sample sizes in these earlier experimental intervention studies were small with low statistical power to detect true effects.

### Current Study

The present training study was therefore set up to test the influence of the writing tool on the acquisition of literacy skills at the letter and word level with various tests in a large sample of kindergarten children (*n* = 147). Given this large sample, we were able to statistically control for the influence of a variety of potentially confounding variables related to prior letter knowledge and precursor skills of literacy acquisition. Using closely matched letter learning games, children were trained by handwriting with a pencil on a sheet of paper (pencil group) or by writing on a digital device. As tablet computers with touchscreens are increasingly popular writing tools in educational settings due to their simplicity of use (Couse and Chen, 2010; Buchegger, 2013), we decided to provide tablet computers as digital writing devices instead of laptop computers, which were mostly used in earlier studies. The tablet computers allowed us to implement two digital training conditions with the same device: In one digital training condition, children wrote the letters by hand on the touchscreen using a special stylus (stylus group). Handwriting training using a stylus on a tablet screen is an interesting comparison condition for traditional handwriting with paper and pencil for two reasons (for discussions, see Alamargot and Morin, 2015; Gerth et al., 2016): Firstly, writing with a stylus on a tablet screen is an option for the classroom, when handwriting, and not typewriting, should be combined with the use of a digital device (Kiefer and Velay, 2016). Secondly, the slippery glass surface of a tablet touchscreen has lower friction than paper and thus provides less sensory feedback for the writer (Guilbert et al., 2019). Writing on a touchscreen might thus increase difficulty of motor control and might lead to disturbed writing movements compared to writing on paper as suggested by several recent studies (Alamargot and Morin, 2015; Gerth et al., 2016; Guilbert et al., 2019). It is therefore important to assess the influence of handwriting with a stylus on a tablet compared with pencil and paper. In the other digital training condition, children were trained to type letters and words using a virtual keyboard on the touchscreen (keyboard group). Although sensory feedback during typing is reduced for virtual compared to real keyboards, we reasoned that an evaluation of such a typing device is practically relevant due to the increasing popularity of tablet computers, also at schools.

We implemented an intense training program for children attending the German kindergarten in the year before elementary school entry (preschool children, age about 4–6 years). Children were trained on 28 training sessions, which were distributed over 7 weeks on 4 days per week. We trained preschool children and not elementary schoolchildren, in order to assess training effects without the influence of previous formal handwriting training as in schoolchildren. Sixteen letters of the German alphabet were trained either by handwriting with a pen on a sheet of paper, by handwriting with a stylus on a tablet screen, or by typing on a virtual computer keyboard. The letter learning games were adopted from our earlier pilot study (Kiefer et al., 2015). However, besides the much larger sample size, training involved more letters and was extended over a longer time interval compared with the pilot study. The different training modes, handwriting with pencil and paper, handwriting with a stylus on a tablet, and typing on a virtual keyboard, were administered to three separate samples of preschool children (pencil: *n* = 49; stylus: *n* = 50; keyboard: *n* = 48) matched for age, sex, phonological awareness, free letter writing, and non-verbal intelligence as possibly confounding variables. Letter recognition, letter reading and letter writing performance were assessed before (T1) and after training (T2) as well as at a follow-up assessment about 4–5 weeks after the training (T3) to measure stability of acquired knowledge. Reading and writing performance of words, which could be composed of the trained 16 letters, were tested only post-training and at follow-up. We also assessed, whether visuo-spatial skills (measured with the FEW-2; Büttner et al., 2008), which might be necessary for letter identification (Palmis et al., 2017), would be specifically improved through handwriting training. We reasoned that handwriting, but not keyboarding requires close attention to the fine-grained visuo-spatial configuration of letters, which have to be reproduced by the motor program. Visuo-spatial skills were therefore tested before and after the intervention. Groups of five to nine children were trained by experimenters in a separate and quiet room of the kindergarten, but as part of the regular kindergarten schedule to obtain a naturalistic learning environment.

If the easiness of the motor program facilitates letter recognition, reading and writing, a superiority of typing training should be observed over handwriting training with pencil or stylus. In contrast, if a meaningful coupling between action and perception facilitates literacy training, handwriting training should be superior to typing training. Furthermore, we expected that handwriting training (pencil or stylus) fosters development of visuo-spatial skills in contrast to keyboard training. This superiority of handwriting training should be larger for writing with a pencil than with a stylus due to the increased sensory feedback children receive, when writing with a pencil on paper.

## Materials and Methods

### Participants

Power calculations using GPower (Erdfelder et al., 1996) indicated that 45 children per group (three groups, in total 135 children) a needed to detect a small interaction effect (*d* = 0.25) between group and time points of assessment (before training, after training, follow up) with a power (1-beta) of 0.90 at a *p* < 0.05. In order to account for a potential drop out, we recruited 173 children from 10 different kindergartens in the area of Ulm, Germany. All children attended the last year of kindergarten prior to school. Twenty children were excluded prior to the beginning of the study based on information in the parent questionnaire. The exclusion criteria were multilingualism (6 children), diagnosis of a developmental, neurological or psychiatric disorder (5 children) and current speech disorder (9 children). All children had normal-or corrected-to-normal vision and normal hearing according to parents’ and educators’ report. Tests for non-verbal intelligence assessed with the CPM (Raven et al., 2010) and phonological awareness assessed with the BISC (Jansen et al., 2002) were administered, in order to exclude children with exceptionally low test scores and additionally to match the participants assigned to the different intervention groups (see below). The BISC is used for the early detection of precursor abilities important for reading and writing acquisition. It covers the ability areas *phonological awareness* (4 tests), *fast retrieval from long-term memory* (3 tests), *phonetic recoding in short-term memory* and *visual attention control* (in each case 1 test). All four tests for phonological awareness (rhyming, syllable-segmenting, phonetic-to-speech and lute-associating) were used in the study to comprehensively assess this ability as an important control variable. If the percentile rank in the CPM was less than 10, or if the child had three or more risk points in the BISC, he or she was excluded from the study (6 children). According to the manual (Jansen et al., 2002), a child receives a risk point for a BISC subtest, when his or her performance can be achieved solely by guessing, i.e., when accuracy is at chance level. The percentile rank in the CPM (Raven et al., 2010) indicates, how many percent of subjects of the same age in the norming sample have achieved the same or a lower test value. It is therefore an age-adjusted score. The CPM manual defines “mental disability” as a percentile rank of 5 or less. By choosing the more conservative cut-off value of 10 and below in the present study, we ensured that no children with intellectual disabilities were included. According to the CPM manual, the familiar IQ norms cannot be meaningfully applied, because no normal distribution of the CPM test scores can be assumed due to developmental leaps in the captured construct. The resulting sample (*N* = 147, 75 female, *M* = 5 years and 10 months; *SD* = 5,2 months) was split in three matched groups, which were assigned to the three different training conditions: handwriting with paper and pencil (*n* = 49, “pencil group”), handwriting with a special stylus on a tablet screen (*n* = 50, “stylus group”) and typing on a virtual keyboard (*n* = 48, “keyboard group”). The variables used for matching were phonological awareness according to BISC (raw scores), prior letter knowledge (raw scores of free letter writing, see below), age, gender and non-verbal intelligence according to CPM (percentile ranks). The matching with these five variables was intended to ensure that the children in three different groups were comparable with regard to these variables. For the purpose of gathering information about prior letter knowledge, each child was given a blank sheet with the task of writing down all the letters they know spontaneously (Kiefer et al., 2015). This task took place before the assignment to the three different training conditions, and all children completed this task with a pencil on a sheet of paper.

During the study, one child (from the keyboard group) decided to leave the training. After the study another child (from the stylus group) was excluded because of too little attendance (less than 30% of the training days present). In the final analysis, the data of 145 children are included. Demographic data for the three different groups are shown in Table 1. Participant groups of the final sample did not differ in the variables used for matching the subsamples: [*F*_*Age*_(2,142) = 0.672, *p* = 0.512; $\chi_{S\,e\,x}^{2}$(2) = 2.710, *p* = 0.258; *F*_*Phonological  awareness*_(2,142) = 0.620, *p* = 0.539; *F*_*Free  letter  writing*_(2,142) = 0.310, *p* = 0.734; *F*_*Nonverbal  intelligence*_ (2,142) = 0.112, *p* = 0.894].

**TABLE 1:** Descriptive data of the 145 children on the matching and control variables across the three different conditions.

**Variable**	**Total (*N* = 145) Mean(*SD*) [Min – Max]**	**Pencil (*N* = 49) Mean (*SD*) [Min – Max]**	**Stylus (*N* = 49) Mean (*SD*) [Min – Max]**	**Keyboard (*N* = 47) Mean (*SD*) [Min – Max]**
Age (years; months)	5;97 (0;53) [4;10 – 6;11]	5;98 (0;59) [4;11 – 6;11]	5;10 (0;48) [5;00 –6;70]	5;90 (0;50) [4;10 – 6;70]
Sex	75 female, 70 male	29 female, 20 male	26 female, 23 male	20 female, 27 male
Phonological awareness (raw score)	36.34 (3.13) [25 – 40]	35.94 (3.63) [25 – 40]	36.55 (2.89) [28 – 40]	36.55 (2.81) [26 – 40]
Free letter writing (raw score)	8.38 (3.45) [1 – 24]	8.10 (2.90) [3 – 18]	8.39 (3.56) [1 – 19]	8.66 (3.89) [3 – 24]
Non-verbal intelligence (percentile rank)	65.32 (24.49) [11 – 100]	65.04 (25.75) [11 – 100]	64.33 (24.94) [11 – 98]	66.66 (23.08) [11 – 98]
Letter reading (raw score at T1)	8.37 (5.11) [0–16]	8.24 (5.48) [0–16]	7.88 (4.57) [0–16]	9.00 (5.30) [0–16]
Visuo-spatial skills (raw score at T1)	15.77 (5.12) [5–25]	15.27 (5.57) [5–25]	15.96 (5.05) [5–23]	16.11 (4.76) [5–24]

As already mentioned above, children in the last year of German kindergarten took part in the study, before they enter elementary school (preschool children). As can be seen in Table 1, the range for the age of the children is from 4 years and 10 months up to 6 years and 7 months. At first glance, this appears to be a wide range. However, this is the usual age range of German preschool children. The matching made the age range comparable across the different groups.

Prior to the study, written informed consent was obtained from the parents of the children. This study was carried out in accordance with the recommendations of the ethics committee of Ulm University. The protocol was approved by the ethics committee of Ulm University (application number 71/16). The parents of all subjects gave written informed consent in accordance with the Declaration of Helsinki.

### Material and Procedures

#### Letter and Word Knowledge Assessment

Writing and reading performance was assessed at the level of individual letters as well as at the level of words. At the level of letters, recognition performance was also measured. In the following, the test procedures are described, and the time points of assessment are indicated. Depending on the tests, there were up to three time points of assessment: Performance data were collected before the training (=T1), immediately after the end of the 7-week training (=T2) and 4–5 weeks after the end of the training (=T3).

##### Letter recognition

**FIGURE 1:**
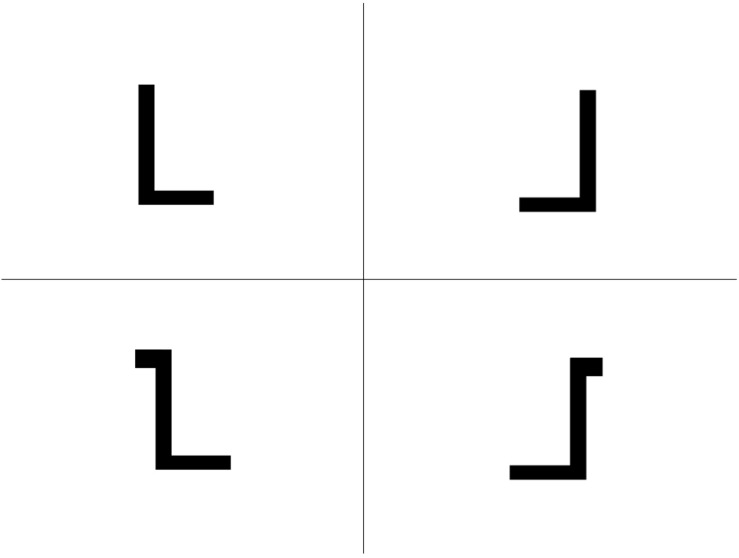
The letter recognition test (example: letter L).

In the letter recognition task, four characters were shown to the children of all training groups on a tablet screen (see Figure 1): One of the trained letters in its correct form and three distractors. The distractors were formed as follows: First distractor: Mirror image of the correct letter or turned upside down image, if mirroring was not possible, e.g., for the letter A. Second distractor: Variation of the correct letter, which was done in three ways: (a) Addition of a line. (b) Deletion of a line or, if (a) and (b) were not possible, changing a rounding (c). The third distractor was generated by mirroring the second distractor or by turning it upside down, if mirroring was not possible. Thus, neither degree of complexity of the character nor its spatial orientation should serve as an indication for the correct solution. Because this standardized variation of the distractors could not be performed for all 16 trained letters (not possible with: O, H, I, M, W) only 11 letters were included in this test (L, A, S, E, K, T, F, U, P, R, J). The items were presented in random order and with variant position of the target in the four-quadrant matrix. The child had the task to touch the correct letter as fast as possible on the screen. The correctness of the response were recorded by the tablet. The child could reach up to 11 points as 11 letters were tested. This test was taken at all time points of assessment (T1, T2, T3).

##### Letter writing

The child was presented with audio recordings of the letter read aloud in its phonetic form by a female voice. The child had to write or to type the respective letter using the assigned writing medium. We opted for the phonetic pronunciation of the letters, since the letter name in German for consonants is formed from two phonemes (e.g., B - BE). This can lead to confusions in the children, when writing the letters. The letters were presented in the following fixed order: O, I, M, T, R, J, F, P, H, K, W, U, E, L, S, A. The letter writing test was administered at all three time points of assessment (T1, T2, T3). As mirror image writing is common in children at this age, in addition to the correct letter, letters in mirrored form were also considered as correct (this applies to the pencil and stylus conditions, but not to the keyboard condition, since no mirroring is possible when typing). However, as it turned out, results did not change, when only correctly written letters were scored. Children could reach between 0 and 16 points in this test.

##### Word reading

During word reading, the child was presented with the words on word cards (each word separately) and was asked to read aloud the word. As the children were not supposed to be able to read words before the training, this test was only performed immediately after the training (T2) and after an interval of about 4–5 weeks after the training (T3). The words used were UHU (Eng.: eagle owl), PAKET (Eng.: package), WELT (Eng.: world), KIWI (Eng.: kiwi fruit), FELS (Eng.: rock). The words were composed of the trained letters. The children received a point for each word if they were able to read the word correctly (that is, pronounced the corresponding phonemes). Children could reach between 0 and 5 points in this test.

##### Word writing

In the word writing test, children were presented with audio recordings of one- or two-syllable concrete words with clear phoneme-to-grapheme correspondence, spoken by a female voice. Each word was repeated three times, the second time the pronunciation was stretched to make it easier for the children to recognize the individual phoneme corresponding to a letter. The children had to write or to type the spoken word. All words were composed of the trained letters (LOK [Eng.: locomotive], KAMEL [Eng.: camel], PILOT [Eng.: pilot], HEFT [Eng.: booklet], SALAT [Eng.: salad]). In order to be able to continuously evaluate children’s abilities, also partially correct solutions were considered for scoring. For that reason, the mean proportion of correctly written letters at the correct position in the word per word was taken as test score (mean percentage of correct letters/number of letters of a word). Again, mirrored letters, as described above, were considered as correct. Due to its complexity, this test was administered post training (T2) and at follow-up (T3) only.

#### Additional Control Variables

##### Letter reading

The ability of children to read single letters was assessed as an important control variable (e.g., Foulin, 2005; Molfese et al., 2006). In particular, performance at T1 was used to account for existing letter knowledge. Children were successively presented individually with all 26 letters of the alphabet on cards (order: L, I, O, A, M, S, T, E, R, U, D, N, F, B, Z, W, H, K, P, G, V, C, J, Y, X, Q). The children were instructed to say “stop” and name the letter aloud whenever they had the feeling of knowing the letter name (if correct: 1 point). We use this type of approach because it avoids frustration in children with only little letter knowledge before the training. Note that preschool children in Germany do not receive formal literacy training. Only the 16 letters that were part of the training set were considered for analysis. The children could reach a maximum of 16 points in this test. This test was performed at T1, T2 and T3. Performance of letter reading before training (T1) was used to control for already existing letter knowledge. As we decided to use letter reading as control variable, we did not compare performance in letter reading before and after the intervention. However, an analysis of all three time points of assessment with letter writing as control variable did not yield significant group differences (results not shown). The descriptive data of letter reading at T1 is shown in Table 1. The descriptive data for the other two time points of assessment (T2 and T3) are reported in the Supplementary Table 1.

##### Visuo-spatial skills

Visuo-spatial perceptual skills were also tested for the following two reasons: Firstly, visuo-spatial skills before the training (raw score at T1, see Table 1) served as control variable, because they might be important precursor abilities for reading and writing acquisition (Palmis et al., 2017). Secondly, visuo-spatial skills might be improved through training. This variable was therefore also included in the analyses assessing training effects (raw score, from T1 to T2).

Subtest 2 of the FEW-2 (Büttner et al., 2008) was used for this purpose. The FEW-2 subtest 2 “Position in Space” captures the ability to compare two figures in terms of common features or spatial positions (manual p. 45). In this test, the child is presented with increasingly complex shapes as probes. The child has to subsequently point to the target shape identical with the probe among an increasing number of distractors. To perform this test, the child has to compare the spatial layout of a probe figure with distractor figures and to find a second figure with the same layout under a number of distractors. Distractors can be rotated or mirrored versions of the original drawing. In the first half of the test, simple two-dimensional figures are presented, whereas in the second half of the test three-dimensional objects or a number of overlapping objects are used as probes and distractors, thus increasing the difficulty of the spatial relations between the parts of the figure. For each correct response, the child received one point and could reach a total of 25 points. This test was performed before (T1) and after training (T2).

#### Letter and Word Training

As an experimental intervention, a standardized training was developed based on the procedure of our earlier pilot study, in which less letters were trained over a short period of time (Kiefer et al., 2015). Across 7 weeks, 16 uppercase letters of the German alphabet (L, A, S, E, O, K, T, F, H, U, P, I, M, W, R, J) and 12 words, which can be composed of these letters, were trained in 28 training sessions (four times a week for 25 min each) by means of a repeating sequence of different letter games. Criteria for letter selection were a clear symbol-sound correspondence and a high frequency of use in written German language. Ambiguous letters referring to two or more different phonemes were excluded (e.g., letter V in German language). For pairs of letters that differed only slightly in phonology (e.g., B-P, G-K), only one of each pair was chosen, in order to avoid phonological confusions. The size of each training group was five to nine children. The training was conducted in a standardized manner. For this purpose, a detailed training manual was created. This manual contained verbatim formulations for instructions, additional assistance and feedback to the children as well as pedagogical hints. The trainers had pedagogical experience with children before the training. They were additionally trained before the training both in content of the training and with regard to the interactions with the children. The standardization and quality of the trainer-child interactions were confirmed by means of video recordings. For this purpose, the trainer was filmed during the training units in fixed periods of time. These recordings were checked by the supervisor (an educator and psychologist) for compliance with the training manual. These measures ensured standardization with regard to the intensity of the training in each group (e.g., numbers of letters written), but also with regard to the extent and type of feedback or assistance provided to the children, even in comparably large groups of 5–9 children.

**FIGURE 2:**
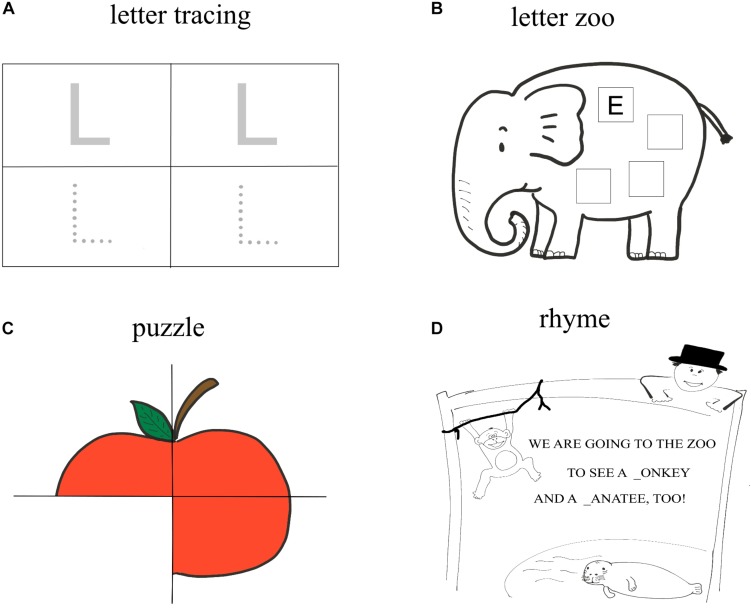
Overview of the training task used for written language training in kindergarten children. The tasks were the same for all three trainings groups (pencil group, stylus group and keyboard group). They differed only with regard to the writing mode (writing with a pencil on a paper vs. writing with a stylus in the tablet surface vs. typing on a digital keyboard). Top left **(A)** = letter tracing. Top right **(B)** = letter zoo. Bottom left **(C)** = puzzle. Bottom right **(D)** = rhyme completion.

The training procedure was identical for all three training conditions, except for the writing medium. In the first week, the children learned four letters, in the weeks 2–7 they were taught two additional letters. From the second week on, children practiced words, which could be composed of the letters learned (training week 2: one word, training week 3–6: two words, training week 7: three words). In every training week, one new letter was introduced on the first day and a second one on the second day of training. On the third day, the two new letters were practiced conjointly in an alternating fashion. On the fourth day, all the previously trained letters were repeated in letter games. An exception to this procedure was, as already mentioned, week 1. Here, a new letter was introduced on each of the 4 days. These letters were very easy and already familiar to many children (L, A, S, E). The four letters were briefly repeated at the beginning of the second week before the next new letter was introduced. The introduction of a new letter was embedded in an ongoing story. The story was based on a book used for literacy training in elementary schools (Mai et al., 2004). This story was about Lili and Oli, two friends, who miraculously travel through a magical land of letters, where they encounter new letters. Before the children wrote the new letter, the letter was introduced with the 4S method (“show,” “say,” “sound,” “stress”; Weitzman and Greenberg, 2010). First, the printed letter was shown to the child (“show”). Thereafter, the trainer pronounced the letter (in phonetic form; “say”) and requested the children to listen to the letter sound (“sound”). The last step was “stress.” For this, the trainer spoke a word, which starts with the respective letter in a stretched way (e.g., LLLLillllli). During these four steps, the letter was shown to the children as picture on the respective page of the story. After this introduction, children were trained individually with different letter learning games, which are described below (see also Figure 2). Even if some games were played in groups of children (e.g., gremlin, magic potion of the witch), each child individually wrote/typed the letters on its private writing template (on a sheet of paper, on the tablet) so that each child wrote the same number of letters. During the training games, the children received feedback regarding the correctness of their responses from the trainers.

The aim of the training was to stimulate children writing each letter as often as possible. All letters were playfully practiced in the following letter games:

##### Letter tracing

Each child in the handwriting group received a paper with the letter clearly printed (twice) as well as with the letter printed in the form of unconnected dots (twice) (see Figure 2A). The children from the stylus and the keyboard groups saw the same stimuli on the tablet screen. The children had the task to trace the respective letter with the pencil/stylus (pencil and stylus groups) or, in the keyboard group, to search for and type the corresponding letter on the keyboard. Hence, the children in the keyboard group had to find and press the key for the correct letter on the keyboard (the letter was clearly visible on the key) for both the clearly printed template and the dotted template. Each letter was traced/typed twice with the dotted template and twice with clearly printed templates. This game was administered once for the introduction of a new letter.

##### Letter zoo

The children were shown a picture of an animal (see Figure 2B). They were asked to name the animal and to indicate, with which letter its name starts (e.g., elephant, and the letter E). Afterward, the children were asked to write/type the first letter four times on the depicted animal. The children in the pencil group received a piece of paper on which the animal was depicted. The children from the stylus and the keyboard group saw the image of the animal on the screen of the tablet. For each letter, there were three different animals. Two of them were used for the introduction of a new letter (on day one of the training week for the first new letter or on day two of the training week for the second new letter), and one animal on the third day of the training week, when the two new letters from the respective week were repeated conjointly in the same training session in an alternating fashion.

##### Puzzle

In the pencil and the stylus group, each child was given four covert pieces of a puzzle made of paper (see Figure 2C). For the stylus group, a template was opened on the tablet screen, which had the same shape as the concealed puzzle pieces. These puzzle pieces were later used in the task as a template for writing the letter. The children from the keyboard group saw the covert puzzle on the screen of the tablet. The children were told that they should not reveal the puzzle pieces until they wrote/typed the letter to be trained on each piece of the puzzle and the trainer has checked its correctness. If the letter was correct, the children of the pencil and stylus groups turned the puzzle pieces over by hand. In the keyboard group, this was done digitally, when entering the correct letter. If the children did not write the letter correctly, they had one more trial, before the trainer showed the correct answer. The children then were allowed to write/type the correct response and turn the puzzle pieces. For each letter, there were three different puzzles, two of them were used for the introduction of the new letter, and one puzzle on the third day of the training week, when the two new letters from the respective week were repeated.

##### Rhyme completion

In the “rhyme completion” game, each child had a template with a short rhyme. In the pencil group this was on paper, in the keyboard and stylus groups the rhyme was digitally presented on the screen of the tablet. The children were told that the letter to be trained is missing in two places within the rhyme. These places were highlighted with gaps (see Figure 2D). Through this visual hint, the children could fill the gaps with the missing letter without having to be able to read. The children were asked to fill the two gaps with the corresponding letter. If this was done correctly, the trainer read out the complete rhyme. There were two rhymes per letter. One was used when introducing a new letter. The second rhyme was used on the third day of each week for the repetition of the new letters trained in the corresponding week.

##### Letter games for interactive play (gremlin, magic potion of the witch)

On the fourth day of each training week, all previously practiced letters were playfully repeated. All children played together two games in weekly turns. In the first game, *gremlin*, there were cards in the middle of the table. Each of the cards showed an object that started with an already trained letter. The aim of the game was to collect as a group as many cards as possible. Each child was allowed to dice in turns. The dice showed three different pictures. When the dice showed a flower, the child was allowed to reveal a card. The child named the object on the card and the initial letter of the object name. In order “to document,” which cards the group had already collected, each child wrote/typed the first letter of the object name on an empty template. In the pencil group, this was on paper, in the keyboard and stylus groups the template was digitally presented on the screen of the tablet. If the dice showed the gremlin, one of the cards already collected was taken away. When the dice showed a fairy, the fairy took the card from the gremlin and “gave it back” to the children. On this occasion, the children did not write/type the letter, in order to keep the number of letters written/typed in the various groups constant. In the other game, *magic potion of the witch*, there was again a deck with cards placed in the middle of the table. The cards displayed objects, which started with the letters to be trained. There were two cards per letter. One child started by turning two cards from the deck. If the two depicted objects were identical, the child named the object on the card and the initial letter of the object name. This object then was one of the ingredients for the witch potion. In order to write down the “recipe” for the potion, all children wrote/typed the respective letter on a template. If the cards, which were revealed, were different, they were placed back on the bottom of the deck, and the next child took the turn. These games served to repeat the known letters and to stimulate individual letter writing in an entertaining fashion.

##### Word writing and word reading

As already mentioned, not only letters but also words, which can be composed of the trained letters were practiced. Here, the word material was limited to phonetically accurate one- and two-syllable words with a length of 3–5 letters. Words had a clear, direct grapheme-phoneme correspondence and were common words, most likely familiar for the children. The occurrence of different letters in the words was balanced as far as possible. The trained words were: LOK (Eng.: locomotive), SALAT (Eng.: salad), FELS (Eng.: rock), HEFT (Eng.: book), UHU (Eng.: eagle owl), PAKET (Eng.: packet), PILOT (Eng.: pilot), KAMEL (Eng.: camel), WELT (Eng.: world), ROT (Eng.: red), KIWI (Eng.: kiwi), JOJO (Eng.: yo-yo).

In the word reading task, the children were shown the word printed on a sheet of paper. The children were asked to read the word silently on their own. Children, who had an idea of how to pronounce the word, raised their hand and were called by the trainer. The reading attempt of the first child, who gave an answer, was documented, in order to keep track during the course of the training, which child was called on and how the child performed. Over the course of the 7-week training, the trainer made sure that each child was called to give the first answer at least once to actively engage all children in the reading exercise. When all children had made their assumptions about the correct pronunciation of the word, the word was worked out together, letter by letter. For this purpose, the trainer encouraged the children to name the single letters. The trainer called the children. If the answer was right, the trainer encouraged the child to connect the named letters (step by step synthesis of the phonemes): e.g., for salad, S + A = sa, S + A + L = sal and so on. If the answer was false, the trainer called another child to name the single letter. These steps were done, until the word was completed.

In the word writing task, the trainer pronounced the word slowly and repeated it three times. At the second repetition of the word, the word was pronounced very slowly and stretched, in order to make it easier for the children to identify each phoneme corresponding to a letter. After the third repetition, the children were asked to write/type the word individually. Next, the correct solution was worked out together. For this purpose the trainer and the children tried together to hear every single letter out of the word. Children, who had an idea about the correct answer, raised their hands and were called by the trainer. If the answer was right, the children were asked to write down the letter below its first attempt. If the answer was false, the trainer called another child. These steps was done for every letter of the word. This second attempt served to visualize the correct solution. Both the reading and the writing of the 12 words were practiced once per word during the entire course of the training.

### Statistical Analysis

All data preprocessing and data analyses were performed with IBM SPSS Statistics (version 25). The available data were checked (to 100%) for input errors (errors that may occur when transferring data from the paper protocol sheets to the digital data file). Any errors found were documented and corrected.

The dependent variables were checked for implausible values (values that are not possible, e.g., outside the possible range of a scale). For this purpose, if the prerequisite of the distribution was given, all cases were checked, which were ±3 standard deviation from the group mean value. If the prerequisite of the normal distribution was not given, a box plot was created and all values outside the whiskers were checked for plausibility and possible input or survey errors. This check for extreme values showed no abnormalities.

The following statistical approaches were used to test intervention effects on the dependent variables: If the analysis of interest comprised only one time point of assessment, multiple regression was calculated with test scores as dependent variables. If the dependent variable was considered for more than one time point of assessment, linear mixed models (LMMs) were calculated to statistically account for repeated measurement within a child. The calculated models (regression and LMMs) are depicted in Table 2. All linear mixed models included *subject* as a random effects factor with a random intercept. One fixed effect factor for all analyses (regression and LMMs) was *group* (= writing medium during the training). As this factor had three levels (pencil, stylus, keyboard), it was dummy coded. In order to be able to statistically test all interesting comparisons between the three groups, one model was calculated with the pencil group as reference and one model with the keyboard group as reference. The reference group received the value 0 for dummy coding, the contrast group the value 1. As the groups were already matched for phonological awareness, prior knowledge on the letter level (free letter writing), age, gender and non-verbal intelligence these were not included as control variables. However, scores of visuo-spatial skills (T1) as well as letter reading ability (T1) were included as additional control variables in the models for the letter and word tests (regression and LMMs). The control variables were centered (on the overall mean value).

**TABLE 2:** Overview of the calculated models (x, **X**).

**Dependent Variables**	**Learning increment T1 vs. T2**	**Stability T2 vs. T3**	**Learning gain T1 vs. T3**	**Ability T2**
Letter recognition	x	x	**X**	Not calculated
Letter writing	x	x	X	Not calculated
Word reading	–^1^	x	–^1^	**X**
Word writing	–^1^	x	–^1^	**X**
Visuo-spatial skills	**X**	–^2^	–^2^	Not calculated

*T1, assessment before training; T2, assessment after training; T3, assessment 4–5 weeks after training; x, calculated model (see Supplementary Tables 6–12); highlights (**X**): identify models in which a significant group difference was observed; –^1^, dependent variable was not quantified at T1, so the model could not be calculated; –^2^, dependent variable was not quantified at T3, so the model could not be calculated.*

In the LMMs, time point of assessment was included as additional fixed effects factor. As the factor *time point of assessment* had three levels (T1, T2, T3) and as we could not necessarily assume a linear increase of performance over time, only two levels, i.e., time points of assessment, were included in the LMMs. We therefore calculated the following LMMs, which capture different aspects of learning: The increase in learning from T1 to T2 indexes the *learning increment*. The change from T2 to T3 reflects *stability* of learned knowledge after the end of training. Finally, the comparison between T1 and T3 captures the *learning gain* across the entire observation period. Word reading and word writing were only assessed at T2 and T3. For this reason, it was not possible to test possible group differences with regard to learning increment (T1 vs. T2) or learning gain (T1 vs. T3) using LMMs. Instead, possible group differences in test performance at T2 (= *ability*) were statistically assessed for the two word tests using regression models. Furthermore, stability (T2 vs. T3) was calculated with LMMs for the two word tests. Hence, as dependent variables were not available for all time points of assessment (see Table 2), learning increment and learning gain could not be calculated for all dependent variables. Table 2 provides an overview of all comparisons across time points of assessment that were possible with the collected dependent variables. LMMs or a regression approach was used depending on the number of time points of assessment included in the analysis for the respective dependent variable.

In total 22 models were calculated: Eighteen LMMs with respective reference groups (2 ^∗^ 9 = 18) and four regressions with respective reference groups (2 ^∗^ 2 = 4). Due to the complexity of the statistical analyses, we describe in the results section only the results of those models, in which group differences were statistically significant. Results of other models, which did not yield significant group differences are reported in the Supplementary Tables 6–12. The study material is available to interested researchers on request.

## Results

### Correlation of Control Variables With Reading and Writing Performance

The three groups (pencil, stylus, keyboard) were matched as described in Section “Participants” for phonological awareness, prior knowledge on the letter level (free letter writing), age, gender and intelligence. As visuo-spatial skills (T1) and letter reading performance (T1) were not yet available at the time of matching, these variables were considered as further control variables. In order to check the relevance of these additional control variables for literacy acquisition, correlations between the control variables and the dependent variables at the time points after the end of the training were calculated. To increase the statistical power, these correlations were calculated within the entire sample collapsed across the three groups. In order to reduce complexity of these analyses, dependent variables were averaged across T2 and T3. Table 3 shows the correlative relationships between these control variables (visuo-spatial skills and letter reading, both on T1) and the dependent variables of the letters and word tests (letter recognition, letter writing, word reading, word writing). There were statistically significant correlations between all control variables (letter reading and visuo-spatial abilities) and the dependent variables. The correlations calculated separately for the three groups are reported in Supplementary Table 2.

**TABLE 3:** Correlations between dependent variables and control variables. In order to reduce complexity, dependent variables were collapsed across T2 and T3 and across groups.

	**Control variables**	
**Dependent variables**	**Letter reading T1**	**Visuo-spatial skills T1**
Letter recognition	0.21*	0.31**
Letter writing	0.81**	0.33**
Word reading	0.70**	0.25**
Word writing	0.70**	0.27**

T1 = assessment before training; *indicates a significant correlation at *p* < 0.05; **indicate a significant correlation at *p* < 0.01.

### Differential Trainings Effects as a Function of the Writing Medium on Reading and Writing Performance

**FIGURE 3:**
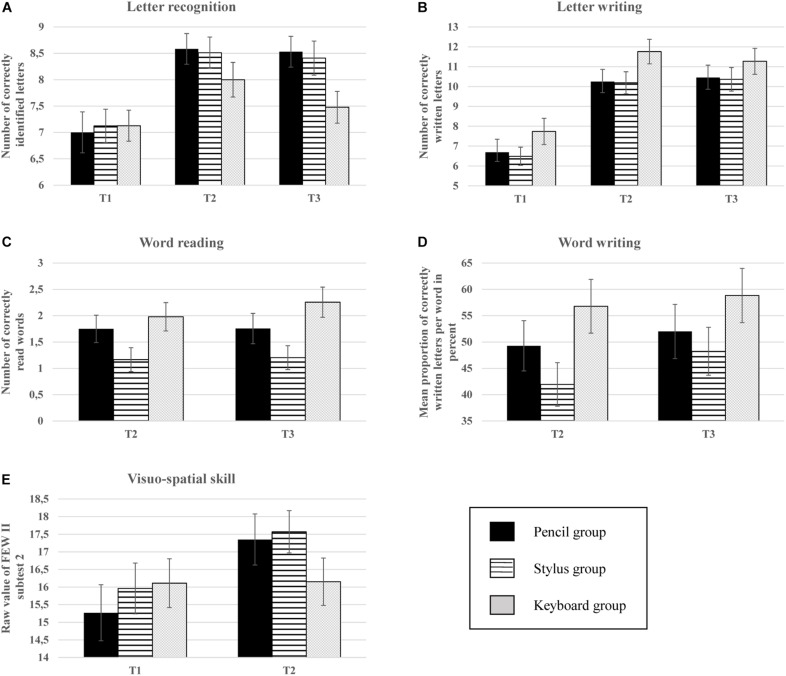
The performance of the children in the different tests (letter recognition, letter writing, word reading, word writing, and visuo-spatial skill) separately for the three trainings groups (pencil group, stylus group, and keyboard group) as a function of time points of assessment (T1, T2, T3). Shown are mean scores (number of correct response: letter recognition, letter writing and word reading and visuo-spatial skill) or mean percentages scores (relative frequency of correct response: word writing). Top left **(A)** = letter recognition. Top right **(B)** = letter writing. Middle left **(C)** = word reading. Middle right **(D)** = word writing. Bottom left **(E)** = visuo-spatial skills.

Performance of the children in the different groups did not differ significantly from each other at T1 in all tests administered at this time (see Figure 3 and in Supplementary Tables 3–5). Word tests were not administered at T1, but most likely, due to the high literacy requirement for word level performance, the children entered the training without relevant previous knowledge. Overall, analyses suggest that performance level before training was comparable across groups.

Results at the different time points of assessment are shown in Figure 3. The descriptive data of all dependent variables, calculated separately for each group are numerically reported in Supplementary Tables 3–5.

All calculated models are shown in Table 2 (see x and X in the Table as symbols for calculated models). The X in bold indicates that a significant group difference was observed in the respective model. In order to reduce complexity, we describe below in the main text only those analyses, in which significant group differences were obtained. The other analyses are reported in the Supplementary Tables 6–12.

#### Letter Recognition (Figure 3A)

In the letter recognition test, the pencil group showed the largest increase from T1 to T2 and the smallest loss from T2 to T3. For this group, if one considers the entire observation period from T1 to T3, the greatest learning gain was obtained (learning increase from T1 to T2 + stability from T2 to T3). Group differences in the learning increase (T1 to T2) and in the learning stability (T2 to T3) were not significant (see Supplementary Tables 6, 7). However, the children from the pencil group showed a significantly larger learning gain (T1 to T3) than the children from the keyboard group. In this comparison the interaction time point of assessment and group was significant. The other group comparisons (pencil group vs. stylus group and keyboard group vs. stylus group) did not yield significant interactions of interest (time point of assessment ^∗^group). In addition, these analyses indicated that children with better visuo-spatial abilities before the training (at T1), showed generally better performance in the letter recognition test (Table 4).

**TABLE 4:** Results of estimated LMMs for the variable letter recognition to T1 vs. T3.

	**Estimate (*SE*)**	***df***	***t*-Score**	***p*-Value**	***CI* 95%**	
					**Lower**	**Upper**
**Reference: pencil group**						
Intercept	7.05 (0.31)	264.44	23.00	< 0.001	6.45	7.65
T3 (Reference = T1)	1.53 (0.39)	137.58	3.90	< 0.001	0.76	2.31
Stylus group	0.07 (0.43)	263.98	0.16	0.877	–0.79	0.92
Keyboard group	0.01 (0.44)	264.03	0.026	0.979	–0.85	0.87
Visuo-spatial skills (at T1)	0.09 (0.03)	144.78	3.13	0.002	0.03	0.15
Letter reading (at T1)	0.05 (0.03)	140.48	1.93	0.056	–0.001	0.11
T3 * stylus group	−0.30(0.56)	139.84	–0.54	0.594	–1.42	0.81
T3 * keyboard group	−1.15(0.56)	139.13	–2.04	0.043	–2.27	–0.03
**Reference: keyboard group**						
Intercept	7.06 (0.31)	263.75	22.78	< 0.001	6.45	7.67
T3 (Reference = T1)	0.38 (0.41)	140.61	0.94	0.350	–0.42	1.18
Stylus group	0.06 (0.44)	263.63	0.13	0.898	–0.80	0.92
Pencil group	−0.01(0.44)	264.03	–0.03	0.979	0.87	0.85
Visuo-spatial skills (at T1)	0.09 (0.03)	144.78	3.13	0.002	0.03	0.15
Letter reading (at T1)	0.05 (0.03)	140.48	1.93	0.056	–0.001	0.11
T3 * stylus group	0.85 (0.57)	141.30	1.48	0.141	–0.28	1.98
T3 * pencil group	1.15 (0.56)	139.13	2.04	0.043	0.034	2.27

#### Letter Writing (Figure 3B)

In the letter writing task, none of the calculated models showed significant group differences. These results are reported in Supplementary Tables 8–10. For completeness of the presentation of the results, descriptive data of this test are shown in Figure 3B.

#### Word Reading (Figure 3C)

Please note that this test was only administered after training at T2 and at the follow-up assessment at T3. As Figure 3C shows, at the descriptive level, the children of the keyboard group read more words (at T2 and T3) than the other two groups (pencil group and stylus group). The statistical examination of the group differences, however, showed only a significantly higher performance of the keyboard group compared with the stylus group at T2 (i.e., ability, see Table 5). The other two group comparisons (keyboard group vs. pencil group and stylus group vs. pencil group) were not significant. Previous knowledge of readings letters (at T1) was a significant predictor of word reading performance.

**TABLE 5:** Results of estimated regression for the variable word reading to T2.

	**Estimate (*SE*)**	***t*-Score**	***p*-Value**
**Reference: pencil group**			
Intercept	1.76 (0.19)	9.49	< 0.001
Stylus group	−0.49(0.26)	–1.88	0.062
Keyboard group	0.08 (0.26)	0.28	0.777
Visuo-spatial skills (at T1)	0.01 (0.02)	0.37	0.710
Letter reading (at T1)	0.23 (0.02)	10.31	< 0.001
**Reference: keyboard group**			
Intercept	1.83 (0.19)	9.77	< 0.001
Stylus group	−0.57(0.26)	–2.16	0.033
Pencil group	−08(4.72)	–0.28	0.777
Visuo-spatial skills (at T1)	0.01 (0.02)	0.37	0.710
Letter reading (at T1)	0.23 (0.02)	10.31	< 0.001

#### Word Writing (Figure 3D)

This test was only administered at T2 and at T3. As can be seen in Figure 3D, the children of the keyboard group typed more correct letters at the correct place of a word than children who wrote by hand (pencil and stylus groups) at T2 and T3. However, only the difference between the keyboard group and the stylus group at the post-test (T2, i.e., ability) was statistically significant (Table 6). This means that directly after the training the children from the keyboard group showed a significantly better performance in writing words than the children from the stylus group. With regard to the control variables taken into account, a significant positive association between letter reading performance at T1 and later writing performance was found (see Table 6).

**TABLE 6:** Results of estimated regression for the variable word writing to T2.

	**Estimate (*SE*)**	***t*-Score**	***p*-Value**
**Reference: pencil group**			
Intercept	49.41 (3.29)	15.00	<0.001
Stylus group	−5.67(4.67)	–1.21	0.227
Keyboard group	4.31 (4.72)	0.91	0.363
Visuo-spatial skills (at T1)	0.21 (0.40)	0.52	0.601
Letter reading (at T1)	4.37 (0.40)	11.07	<0.001
**Reference: keyboard group**			
Intercept	53.71 (3.37)	15.93	<0.001
Stylus group	−9.97(4.72)	–2.11	0.036
Pencil group	−4.31(4.72)	–0.91	0.363
Visuo-spatial skills (at T1)	0.21 (0.40)	0.52	0.601
Letter reading (at T1)	4.37 (0.40)	11.07	<0.001

#### Visuo-Spatial Skills (Figure 3E)

As can be seen in Figure 3E, the children who wrote by hand during training (pencil and stylus group) improved their visuo-spatial skills (from T1 to T2), while the performance of the children of the keyboard group basically remained at the initial level. However, only the difference in improvement from T1 to T2 between the pencil and the keyboard group was statistically significant (interaction T2^∗^keyboard group, reference: pencil group), but neither the difference between the stylus group and the keyboard group nor the difference between the pencil group and the stylus group (Table 7). Note that the pencil group had descriptively (but not significantly) the poorest performance at T1, but the largest learning increment at T2, although performance at T2 was still slightly inferior to the stylus group.

**TABLE 7:** Results of estimated LMMs for the variable visuo-spatial skills to T1 vs. T2.

	**Estimate (*SE*)**	***df***	***t*-Score**	***p*-Value**	***CI* 95%**	
					**Lower**	**Upper**
**Reference: pencil group**						
Intercept	15.27 (0.70)	195.83	21.92	<0.001	13.89	16.64
T2 (Reference = T1)	2.03 (0.58)	139.07	3.52	<0.001	0.89	3.17
Stylus group	0.69 (0.98)	195.83	0.71	0.482	–1.25	2.64
Keyboard group	0.84 (1.00)	195.83	0.85	0.399	–1.12	2.80
T2 ^∗^ stylus group	−0.54(0.82)	139.48	–0.66	0.510	–2.16	1.08
T2 ^∗^ keyboard group	−1.94(0.82)	139.09	–2.36	0.020	–3.57	–0.31
**Reference: keyboard group**						
Intercept	16.11 (0.71)	195.83	22.65	<0.001	14.70	17.51
T2 (Reference = T1)	0.09 (0.59)	139.11	0.15	0.882	–1.08	1.25
Stylus group	−0.15(1.00)	195.83	–0.15	0.883	–2.11	1.82
Pencil group	−0.84(1.00)	195.83	–0.85	0.399	–2.80	1.12
T2 ^∗^ stylus group	1.40 (0.83)	139.49	1.69	0.092	–0.23	3.04
T2 ^∗^ pencil group	1.94 (0.82)	139.09	2.36	0.020	0.31	3.57

## Discussion

The present intervention study investigated the influence of the writing tool during early literacy training on reading and writing performance at the letter and word level in a comparatively large sample of kindergarten children. In three matched groups of children attending the last year of German kindergarten (preschool) reading and writing of 16 letters as well as of short words were trained over a period of 7 weeks. Except for the writing tool, training was kept comparably in all groups. In the first group, children wrote the letters during training with a pencil on a sheet of paper, in the second group children wrote with a stylus on the touchscreen of a tablet computer, whereas in the third group children typed on a virtual keyboard of a tablet computer. Before training (T1), immediately after training (T2) and in a follow-up 4–5 weeks after training (T3), we assessed reading and writing performance at the letter and word level using standardized tests. We also assessed visuo-spatial skills before and after training, in order to test, whether the different training regimens affected cognitive domains other than written language. If the easiness of the motor program supports written language acquisition, typing training should result in improved performance after the training compared with the two handwriting training groups (Calhoun, 1985; Castles et al., 2013; Genlott and Grönlund, 2013; Zheng et al., 2013). In contrast, grounded cognition theories (Lakoff and Johnson, 1999; Barsalou et al., 2003; Gallese and Lakoff, 2005; Pulvermüller, 2005; Kiefer and Pulvermüller, 2012; Kiefer and Velay, 2016; Mangen and Balsvik, 2016) suggest that shaping the form of the letters during handwriting leads to an additional motor memory trace, which facilitates written language acquisition. Accordingly, training in both handwriting groups (pencil and stylus) should be better than in the keyboard training group. However, as writing with a stylus on the slippery surface of the tablet screen is associated with lower friction and greater demands on motor control compared with writing with pencil on a paper (Alamargot and Morin, 2015; Gerth et al., 2016; Guilbert et al., 2019), children trained with the pencil on paper should outperform children trained with the stylus on the tablet.

In contrast to our assumptions, differential effects of the writing medium used during our literacy training program were heterogeneous for handwriting with pencil and keyboarding and seem to support either theoretical stance (easiness of the motor program vs. grounded cognition). Children of the pencil group showed superior performance in letter recognition compared to children of the keyboard training group. Performance of children trained with the stylus on a tablet touchscreen did not differ from the keyboard and the pencil groups. Children in the keyboard group performed better in writing and reading words than children in the stylus group. Performance of children of the pencil group did not significantly differ from the other groups. These results suggest that both, handwriting with pencil and keyboarding is associated with specific advantages. Furthermore, handwriting training with pencil and handwriting training with stylus on a touchscreen did not lead to significantly different performance in reading and writing. It should be also noted that observed effects were small and statistically reliable only in some tests and in specific comparisons across time points of assessment. Contrary to our expectations, handwriting training with stylus and tablet did not show significant superior performance in any test compared with keyboarding. Furthermore, test performance in the pencil group was not superior to training with stylus and tablet, although this result corresponds to earlier work (Patchan and Puranik, 2016).

In line with the easiness of typewriting hypothesis (Genlott and Grönlund, 2013, 2016), in the word writing and reading tests after training, children of the keyboard group showed significantly better performance than the children of the stylus group. However, performance in word writing and reading did not significantly differ between the keyboard and the pencil groups. Hence, the easiness of the motor program associated with typewriting (Calhoun, 1985; Castles et al., 2013; Doughty et al., 2013; Genlott and Grönlund, 2013; Zheng et al., 2013) did not generally facilitate training of word writing or reading compared with handwriting training. Instead keyboarding on the tablet was associated with better writing and reading performance only compared with the group of children, who wrote with a stylus on the tablet screen. We assume that the increased effort in controlling the writing movements on the slippery surface of the tablet (Alamargot and Morin, 2015; Gerth et al., 2016; Guilbert et al., 2019) led to inferior word writing because children had to focus their attention on motor control and had less capacity to retrieve and write the correct letters at the appropriate positions of the word (Genlott and Grönlund, 2013). This deficit was only present at the word, but not at the letter level, presumably due to the greater complexity of word writing compared with letter writing. The inferior performance of the stylus group generalized to word reading, although letter recognition and letter writing performance was comparable to the other groups. One may speculate that the quality of the letters written during the training in the stylus group was poor due to the increased demands on controlling writing movements on the tablet surface. As a consequence, the memory representations of the letters might have been less differentiated, when writing was trained with the stylus. This might lead to difficulties, when strings of letters had to be processed for word reading. As for word writing, group differences were not observed at the easier letter level (letter recognition). Possibly, impoverished memory traces for single letters were still efficiently processed in the stylus group. However, at the word level, when several letters are combined in strings and have to be recognized within the context of other letters, deficits in the stylus group might emerge, possibly due to higher competition between letters due to crowding (Huckauf and Heller, 2004). Again as for the writing task, this interpretation is speculative and deserves further investigations. Unfortunately, we did not save the written letters of the stylus group on the children’s training tablets so that the quality of writing could not be assessed. This interpretation of the poor word reading performance in the stylus group is therefore only tentative and deserves future investigations.

In partial support of grounded cognition theories (Lakoff and Johnson, 1999; Barsalou et al., 2003; Gallese and Lakoff, 2005; Pulvermüller, 2005; Kiefer and Pulvermüller, 2012; Kiefer and Velay, 2016; Mangen and Balsvik, 2016), handwriting with pencil and paper had beneficial effects on letter recognition compared with the keyboard training group. Regarding the learning gain (learning increase from T1 to T2 + stability from T2 to T3), letter recognition performance trained by writing with the pencil was superior compared to the keyboard group. Immediately, after the training at T2, the pencil group showed descriptively better performance than the keyboard group, but the group difference in the learning increment was not statistically reliable. Our results therefore indicate that letter knowledge relevant for letter recognition is more endurable, when the letters were trained by handwriting with pencil compared with a keyboard writing training. Our outcomes are therefore generally in line with earlier work (Naka, 1998; Longcamp et al., 2005, 2008), demonstrating better letter recognition or memory after handwriting training using pencil and paper compared with keyboarding training. Most likely, in line with grounded cognition theories, memory traces representing the precise shape of the letters are more stable if a corresponding motor memory trace supports the visual memory trace. Although a similar tendency was also observed for the stylus vs. keyboard group comparison, this effect was smaller and not significant.

Besides letter recognition, handwriting training with pencil improved visuo-spatial skills compared with typing training as shown by the FEW-2 test performance (Büttner et al., 2008) after the training. This indicates that handwriting training with pencil has not only beneficial effects within the domain of written language acquisition, but also contributes to the development of cognitive skills outside the language domain. Handwriting requires precise memorizing the visuo-spatial layout of the letters, which has to be transformed to a motor program from memory (Palmis et al., 2017). Handwriting training thus fosters learning to discriminate and to memorize fine-grained visuo-spatial configurations. This is relevant also for distinguishing stimuli outside the letter domain. Hence, handwriting training can increase visuo-spatial skills in general. In contrast to the improvement of visuo-spatial skills through training in the pencil group, in the keyboard group the level of visuo-spatial skills was comparable before and after the training and thus did not improve. The absence of an improvement in visuo-spatial skills in the keyboard group most likely can be accounted for by the reduced affordances of typing with respect to visuo-spatial discriminations. During typing, the letters can be permanently seen on the keys as visual cues. Therefore, in typing training there is no need to differentiate the letters on a fine-grained basis and to memorize their precise visuo-spatial layout. It is sufficient to establish a coarse memory trace, which can be compared against the letters shown on the keys. As typing draws less on precise visuo-spatial processing compared with handwriting, children in the keyboard group did not improve their visuo-spatial skills during the training. In the stylus training group, improvement of visuo-spatial test performance was descriptively larger than in the keyboard group, but this difference did not reach significance due to the small effect size. As already discussed above, handwriting with a stylus on a slippery tablet surface imposes high demands on motor control and might incur the quality of the established visual and motor memory traces. These particular difficulties associated with writing on a tablet using the stylus most likely reduce the beneficial effects of handwriting for letter recognition and for visuo-spatial skills compared with handwriting using a pencil on a paper.

Contrary to our expectations and to earlier studies (Cunningham and Stanovich, 1990; Kiefer et al., 2015), handwriting training with pencil did not result in superior word writing and word reading performance compared with typing training. Instead, reading and writing performance after training was comparable in both groups similar to other earlier work (Vaughn et al., 1992; Ouellette and Tims, 2014). Apparently, the influence of handwriting training with pencil vs. keyboard training on reading and writing performance is very heterogeneous. However, it should be noted that none of the available studies including the present one obtained evidence in favor of keyboarding, when compared with writing training using a pencil. Keyboarding was only superior to handwriting in our study, when handwriting with the stylus was the comparison condition (see the discussion above). Several factors might explain the heterogeneous findings at the word level: Firstly, reading and writing performance at the word level is typically low and close to floor, in particular when kindergarten children are tested. This reduces the possibility to find any intervention effects because of lacking variability. Secondly, as many intervention studies focused on kindergarten children, only a few words (e.g., 3–5 words) could be tested. Therefore, tests at the word level included only a few trials so that reliability of the assessment is rather poor. Thirdly, there are differences in the procedures across studies such as training duration, test procedure and test material. For instance, in the present study, children were trained for 7 weeks, whereas in our earlier study children were only trained for 4 weeks (Kiefer et al., 2015). It is possible that children might be better acquainted with using the keyboard, when training is longer, thereby diminishing initial beneficial effects of handwriting with pencil.

When interpreting our results, the following limitations should be considered: (i) Effect sizes were generally low despite our relatively large sample size of about 50 children per intervention group, indicating that the influence of the writing tool on reading and writing performance as well as on visuo-spatial skills is not large at the group level. Considerable interindividual variation, caused e.g., by inattention of the child during test or by interindividual developmental trajectories, might mask effects at the group level. (ii) The training period in the present study covered 7 weeks, and the entire observation period including the follow-up test encompassed 14–15 weeks. Although both study intervals were much longer than in previous intervention studies, which covered a few days until 4 weeks, our results are not informative with regard to long-term effects of the writing medium over years. Intervention studies capturing at least the duration of elementary school would be necessary, in order to be able to draw safe conclusions about long-term effects of the writing tool on literacy acquisition as well as on general cognitive development. (iii) Performance in the tasks at the word level was generally quite low indicating that even a training over 7 weeks is not sufficient to obtain a high performance level in largely preliterate kindergarten children. (iv) Although a relatively large set of 16 letters was trained in the present study, potential variation in the dependent measures of the word reading and writing tasks as well as their reliability was quite low, because only a few words could be tested in this strenuous and time-consuming task for these young children. (v) In order to avoid switch costs, if children used a different writing tool at test than at training, we decided to test all children’s writing performance with the same writing tool as used during training. However, to get a more detailed picture how letter and word knowledge acquired with a specific writing tool is expressed, when using a different writing tool, the keyboard group could write in a future study with pencil or stylus and vice versa. (vi) The present results only mirror the current state of digital writing technology. In particular, future technological developments might lead to paper-like tablet surfaces, which render writing with a stylus on a tablet computer comparable to writing with pencil on paper with regard to friction and sensory feedback.

## Conclusion

In partial support of grounded cognition theories (Lakoff and Johnson, 1999; Barsalou et al., 2003; Gallese and Lakoff, 2005; Pulvermüller, 2005; Kiefer and Pulvermüller, 2012; Kiefer and Velay, 2016; Mangen and Balsvik, 2016), kindergarten children trained to write letters with pencil and paper showed superior performance in letter recognition and had improved visuo-spatial skills compared with keyboard training. Performance of children, who were trained with a stylus on a touchscreen, did not differ significantly from the other groups. In letter writing as well as in word writing or reading, handwriting training with pencil and writing training with keyboard did not significantly differ. Keyboard training, however, resulted in superior performance in word writing and reading compared with handwriting training with a stylus on the screen of a tablet computer. Most likely, handwriting with a stylus on a slippery tablet surface imposes high demands on motor control, produces poorly written letters, and thus might compromise the quality of motor and visual memory traces. Because of these motor control difficulties, reading and writing performance of children trained with the stylus on the tablet was inferior to performance of the keyboard training group, where typing imposes low demands on motor control. Despite assumed differences in demands on motor control, performance following handwriting training with pencil and with stylus was statistically comparable in all tests. Given the strengths and weaknesses identified for each writing tool, the results of our study may help to contribute to an understanding, which writing tool should be preferred for literacy training in elementary school. Our results suggest that handwriting with pencil fosters acquisition of letter knowledge and improves visuo-spatial skills compared to keyboarding. At least given the current technological state of tablet touchscreens and styluses, writing with a stylus on a touchscreen of a tablet computer seems to be the least favorable writing tool: Writing training with a stylus on a tablet led to inferior reading and writing performance at the word level compared with keyboarding. At the same time, the beneficial effects of handwriting training on letter recognition and visuo-spatial skills compared with keyboarding were less pronounced compared with writing with a pencil. Technical developments could yield tablet surfaces, in which writing with a stylus is associated with similar friction and provides comparable sensory feedback as in writing with pencil on paper. Future controlled training studies covering a more extended observation period over months or years are needed to allow conclusions about long-term effects of the writing tool on literacy acquisition as well as on general cognitive development.

## Data Availability Statement

The datasets generated for this study are available to interested researchers on request to the corresponding author.

## Ethics Statement

The studies involving human participants were reviewed and approved by the Ethics committee of Ulm University, Ulm, Germany. Written informed consent to participate in this study was provided by the participants’ legal guardian/next of kin.

## Author Contributions

PA, MK, SW, CM, and NB-S designed the present study. CM, SW, NB-S, and SB collected the data. SW and CM analyzed the data. CM and MK wrote the first draft of the manuscript. All authors commented on previous versions of the manuscript, read and approved the manuscript.

## Conflict of Interest

The authors declare that the research was conducted in the absence of any commercial or financial relationships that could be construed as a potential conflict of interest.

## References

[B1] Alamargot D. Morin M.-F. (2015). Does handwriting on a tablet screen affect students’ graphomotor execution? A comparison between grades two and nine. *Hum. Mov. Sci.* 44 32–41. 10.1016/j.humov.2015.08.011 26298215

[B2] Arndt P. A. (2016). Computer usage for learning how to read and write in primary school. *Trends Neurosci. Educ.* 5 90–98. 10.1016/j.tine.2016.07.003

[B3] Barsalou L. W. Simmons W. K. Barbey A. K. Wilson C. D. (2003). Grounding conceptual knowledge in modality-specific systems. *Trends Cogn. Sci.* 7 84–91. 10.1016/S1364-6613(02)00029-23 12584027

[B4] Buchegger B. (2013). *Unterrichtsmaterial Safer Internet im Kindergarten.* Wien: ÖIAT Österreichisches Institut für angewandte Telekommunikation.

[B5] Büttner G. Dracheneder W. Schneider W. Weyer K. (2008). *FEW-2. Frostigs Entwicklungstest der visuellen Wahrnehmung -2.* Göttingen: Hogrefe.

[B6] Calhoun M. L. (1985). Typing contrasted with handwriting in language arts instruction for moderately mentally-retarded students. *Educ. Train. Mental Retard. Dev. Disabil.* 20 48–52.

[B7] Castles A. McLean G. M. Bavin E. Bretherton L. Carlin J. Prior M. (2013). Computer use and letter knowledge in pre-school children: a population-based study. *J. Pediatr. Child Health* 49 193–198. 10.1111/jpc.12126 23437778

[B8] Couse L. J. Chen D. W. (2010). A tablet computer for young children? Exploring its viability for early childhood education. *J. Res. Technol. Educ.* 43 75–96. 10.1080/15391523.2010.10782562

[B9] Cunningham A. E. Stanovich K. E. (1990). Early spelling acquisition: writing beats the computer. *J. Educ. Psychol.* 82 159–162. 10.1037/0022-0663.82.1.159

[B10] Doughty T. T. Bouck E. C. Bassette L. Szwed K. Flanagan S. (2013). Spelling on the fly: investigating a pentop computer to improve the spelling skills of three elementary students with disabilities. *Assist. Technol.* 25 166–175. 10.1080/10400435.2012.743491 24020155

[B11] Erdfelder E. Faul F. Buchner A. (1996). GPower: a general power analysis program. *Behav. Res. Methods Instr. Comput.* 28 1–11. 10.3758/bf03203630

[B12] Foulin J. N. (2005). Why is letter-name knowledge such a good predictor of learning to read? *Read. Writ.* 18 129–155. 10.1007/s11145-004-5892-5892

[B13] Gallese V. Lakoff G. (2005). The brain’s concepts: the role of the sensory-motor system in conceptual knowledge. *Cogn. Neuropsychol.* 22 455–479. 10.1080/02643290442000310 21038261

[B14] Genlott A. A. Grönlund Å (2013). Improving literacy skills through learning reading by writing: the iWTR method presented and tested. *Comput. Educ.* 67 98–104. 10.1016/j.compedu.2013.03.007

[B15] Genlott A. A. Grönlund Å (2016). Closing the gaps - Improving literacy and mathematics by ict-enhanced collaboration. *Comput. Educ.* 99 68–80. 10.1016/j.compedu.2016.04.004

[B16] Gerth S. Klassert A. Dolk T. Fliesser M. Fischer M. H. Nottbusch G. (2016). Is handwriting performance affected by the writing surface? Comparing preschoolers’, second graders’, and adults’ writing performance on a tablet vs. paper. *Front. Psychol.* 7:1308. 10.3389/fpsyg.2016.01308 27672372 PMC5018499

[B17] Guilbert J. Alamargot D. Morin M. F. (2019). Handwriting on a tablet screen: role of visual and proprioceptive feedback in the control of movement by children and adults. *Hum. Mov. Sci.* 65 30–41. 10.1016/j.humov.2018.09.001 30219272

[B18] Gut J. Reimann G. Grob A. (2012). Kognitive, sprachliche, mathematische und sozial-emotionale Kompetenzen als Prädiktoren späterer schulischer Leistungen. *Zeitschrift für Pädagogische Psychologie* 26 213–220. 10.1024/1010-0652/a000070

[B19] Helbig H. B. Graf M. Kiefer M. (2006). The role of action representations in visual object recognition. *Exp. Brain Res.* 174 221–228. 10.1007/s00221-006-0443-5 16636796

[B20] Herzig B. Grafe S. (2006). *Digitale Medien in der Schule: Standortbestimmung und Handlungsempehlungen für die Zukunft.* Bonn: Deutsche Telekom AG.

[B21] Heuer H. (2016). Technologies shape sensorimotor skills and abilities. *Trends Neurosci. Educ.* 5 121–129. 10.1016/j.tine.2016.06.001

[B22] Huckauf A. Heller D. (2004). On the relations between crowding and visual masking. *Percept. Psychophys.* 66 584–595. 10.3758/bf03194903 15311658

[B23] James K. H. Atwood T. P. (2008). The role of sensorimotor learning in the perception of letter-like forms: tracking the causes of neural specialization for letters. *Cogn. Neuropsychol.* 26 91–110. 10.1080/02643290802425914 18830859

[B24] James K. H. Engelhardt L. (2012). The effects of handwriting experience on functional brain development in pre-literate children. *Trends Neurosci. Educ.* 1 32–42. 10.1016/j.tine.2012.08.001 25541600 PMC4274624

[B25] James K. H. Gauthier I. (2006). Letter processing automatically recruits a sensory-motor brain network. *Neuropsychologia* 44 2937–2949. 10.1016/j.neuropsychologia.2006.06.026 16920164

[B26] Jansen H. Mannhaupt G. Marx H. Skowronek H. (2002). *Bielefelder Screening zur Früherkennung Von Lese-Rechtschreibschwierigkeiten (BISC).* Göttingen: Hogrefe, Verlag für Psychologie.

[B27] Kiefer M. Pulvermüller F. (2012). Conceptual representations in mind and brain: theoretical developments, current evidence and future directions. *Cortex* 48 805–825. 10.1016/j.cortex.2011.04.006 21621764

[B28] Kiefer M. Schuler S. Mayer C. Trumpp N. M. Hille K. Sachse S. (2015). Handwriting or typewriting? The influence of pen- or keyboard-based writing training on reading and writing performance in preschool children. *Adv. Cogn. Psychol.* 11 136–146. 10.5709/acp-0178-177 26770286 PMC4710970

[B29] Kiefer M. Sim E.-J. Helbig H. B. Graf M. (2011). Tracking the time course of action priming on object recognition: evidence for fast and slow influences of action on perception. *J. Cogn. Neurosci.* 23 1864–1874. 10.1162/jocn.2010.21543 20617882

[B30] Kiefer M. Sim E.-J. Liebich S. Hauk O. Tanaka J. W. (2007). Experience-dependent plasticity of conceptual representations in human sensory-motor areas. *J. Cogn. Neurosci.* 19 525–542. 10.1162/jocn.2007.19.3.525 17335399

[B31] Kiefer M. Trumpp N. M. (2012). Embodiment theory and education: the foundations of cognition in perception and action. *Trends Neurosci. Educ.* 1 15–20. 10.1016/j.tine.2012.07.002

[B32] Kiefer M. Velay J.-L. (2016). Writing in the digital age. *Trends Neurosci. Educ.* 3 77–81. 10.1016/j.tine.2016.07.008

[B33] Lakoff G. Johnson M. (1999). *Philosophy in the Flesh: The Embodied Mind and Its Challenge to Western Thought.* New York, NY: Basic Books.

[B34] Longcamp M. Anton J. L. Roth M. Velay J. L. (2003). Visual presentation of single letters activates a premotor area involved in writing. *Neuroimage* 19 1492–1500. 10.1016/S1053-8119(03)00088-80 12948705

[B35] Longcamp M. Boucard C. Gilhodes J. C. Anton J. L. Roth M. Nazarian B. (2008). Learning through hand- or typewriting influences visual recognition of new graphic shapes: behavioral and functional imaging evidence. *J. Cogn. Neurosci.* 20 802–815. 10.1162/jocn.2008.20504 18201124

[B36] Longcamp M. Hlushchuk Y. Hari R. (2011). What differs in visual recognition of handwritten vs. printed letters?. An fMRI study. *Hum. Brain Mapp.* 32 1250–1259. 10.1002/hbm.21105 20669164 PMC6870258

[B37] Longcamp M. Zerbato-Poudou M. T. Velay J. L. (2005). The influence of writing practice on letter recognition in preschool children: a comparison between handwriting and typing. *Acta Psychol.* 119 67–79. 10.1016/j.actpsy.2004.10.019 15823243

[B38] Mai M. Fritz M. Reddig-Korn B. (2004). *Das Zauberalphabet. Kinderbuchfibel.* Leipzig: Klett.

[B39] Mangen A. Balsvik L. (2016). Pen or keyboard in beginning writing instruction? Some perspectives from embodied cognition. *Trends Neurosci. Educ.* 5 99–106. 10.1016/j.tine.2016.06.003

[B40] Mangen A. Velay J.-L. (2010). “Digitizing literacy: reflections on the haptics of writing,” in *Advances in Haptics*, ed. Zadeh M. H., (Rijeka: InTech), 385–402.

[B41] Marquardt C. Diaz Meyer M. Schneider M. Hilgemann R. (2016). Learning handwriting at school - A teachers’ survey on actual problems and future options. *Trends Neurosci. Educ.* 5 82–89. 10.1016/j.tine.2016.07.001

[B42] Molfese V. J. Beswick J. Molnar A. Jacobi-Vessels J. (2006). Alphabetic skills in preschool: a preliminary study of letter naming and letter writing. *Dev. Neuropsychol.* 29 5–19. 10.1207/s15326942dn2901_2 16390286

[B43] Naka M. (1998). Repeated writing facilitates children’s memory for pseudocharacters and foreign letters. *Mem. Cogn.* 26 804–809. 10.3758/Bf03211399 9701971

[B44] Ouellette G. Tims T. (2014). The write way to spell: printing vs. typing effects on orthographic learning. *Front. Psychol.* 5:117. 10.3389/fpsyg.2014.00117 24592247 PMC3923165

[B45] Palmis S. Danna J. Velay J. L. Longcamp M. (2017). Motor control of handwriting in the developing brain: a review. *Cogn. Neuropsychol.* 34 187–204. 10.1080/02643294.2017.1367654 28891745

[B46] Patchan M. M. Puranik C. S. (2016). Using tablet computers to teach preschool children to write letters: exploring the impact of extrinsic and intrinsic feedback. *Comput. Educ.* 102 128–137. 10.1016/j.compedu.2016.07.007

[B47] Pulvermüller F. (2005). Brain mechanisms linking language and action. *Nat. Rev.* 6 576–582. 10.1038/nrn1706 15959465

[B48] Radesky J. S. Schumacher J. Zuckerman B. (2015). Mobile and interactive media use by young children: the good, the bad, and the unknown. *Pediatr. Perspect.* 135 1–3. 10.1542/peds.2014-2251 25548323

[B49] Raven J. C. Raven J. Court J. H. (2010). *CPM Coloured Progressive Matrices.* Frankfurt: Pearson Assessment & Information.

[B50] Sim E. J. Helbig H. B. Graf M. Kiefer M. (2014). When action observation facilitates visual perception: activation in visuo-motor areas contributes to object recognition. *Cereb. Cortex* 25 2907–2918. 10.1093/cercor/bhu087 24794918

[B51] Soden-Fraunhofen R. V. Sim E.-J. Liebich S. Frank K. Kiefer M. (2008). Die Rolle der motorischen Interaktion beim Erwerb begrifflichen Wissens: eine Trainingsstudie mit künstlichen Objekten. *Zeitschrift für Pädagogische Psychologie* 22 47–58. 10.1024/1010-0652.22.1.47

[B52] Spitzer M. (2015). Digital genial? Mit dem, Ende der Kreidezeit“ bleibt das Denken auf der Strecke. *Nervenheilkunde* 34 9–16. 10.1055/s-0038-1627558

[B53] Sulzenbrück S. Hegele M. Heuer H. Rinkenauer G. (2010). Generalized slowing is not that general in older adults: evidence from a tracing task. *Occup. Ergonom.* 9 111–117. 10.3233/OER-2010-2176

[B54] Sulzenbrück S. Hegele M. Rinkenauer G. Heuer H. (2011). The death of handwriting: secondary effects of frequent computer use on basic motor skills. *J. Mot. Behav.* 43 247–251. 10.1080/00222895.2011.571727 21598156

[B55] Vaughn S. Schumm J. S. Gordon J. (1992). Early spelling acquisition: does writing really beat the computer? *Learn. Disabil. Q.* 15 223–228. 10.2307/1510245

[B56] Vinci-Booher S. James T. W. James K. H. (2016). Visual-motor functional connectivity in preschool children emerges after handwriting experience. *Trends Neurosci. Educ.* 5 107–120. 10.1016/j.tine.2016.07.006

[B57] Weitzman E. Greenberg J. (2010). *ABC and Beyond: Building Emergent Literacy in Early Childhood Settings.* Toronto, ON: Hanen Centre.

[B58] Zheng B. B. Warschauer M. Farkas G. (2013). Digital writing and diversity: the effects of school laptop programs on literacy processes and outcomes. *J. Educ. Comput. Res.* 48 267–299. 10.2190/Ec.48.3.A

