# Supplementary material for: Literacy Training of Kindergarten Children With Pencil, Keyboard or Tablet Stylus: The Influence of the Writing Tool on Reading and Writing Performance at the Letter and Word Level
Source: Front Psychol. 2020 Jan 22;10:3054. doi: 10.3389/fpsyg.2019.03054 (PMC6987467; doi:10.3389/fpsyg.2019.03054)
Supplement: Supplementary file 1 [file Table_1.pdf]

Literacy training of kindergarten children with pencil, keyboard or tablet stylus: The influence of the writing tool on reading and writing performance at the letter and word level

Carmen Mayer[1,2], Stefanie Wallner[1], Nora Budde-Spengler[1,3], Sabrina Braunert[1], Petra A. Arndt[1§], and Markus Kiefer[2*§]

[1]Transfer Centre for Neuroscience and Education (ZNL), Ulm University, Ulm, Germany

[2]Section for Cognitive Electrophysiology, Department of Psychiatry, Ulm University, Ulm, Germany

[3]Department of German studies, Catholic University of Eichstätt-Ingolstadt, Eichstätt, Germany

[§]Both authors contributed equally to this work

Supplementary Material

1 Supplementary Tables

Supplementary Table 1.

Results of the letter reading task at the different time points of assessment

**Letter reading**	**T2**<br>Mean (*SD*)<br>[Min – Max]	**T3**<br>Mean (*SD*)<br>[Min – Max]
Total	11.70 (4.31)<br>[1 – 16]	11.91 (4.31)<br>[1 – 16]
Pencil	11.54 (4.17)<br>[2 – 16]	11.73 (4.27)<br>[2 – 16]
Stylus	11.55 (4.08)<br>[2 – 16]	11.75 (4.07)<br>[2 – 16]
Keyboard	12.00 (4.75)<br>[1 – 16]	12.28 (4.66)<br>[1 – 16]

Note. T2 = assessment after training; T3 = assessment 4-5 weeks after training;

Supplementary Table 2.

Correlations of the dependent variables averaged across T2 and T3 with the control variables, separately for the different training groups

	**Control variables**					
	Letter reading *T1*			***Visuo-spatial skills*** *T1*		
**Dependent variables**	***Pencil***	***Stylus***	***Keyboard***	***Pencil***	***Stylus***	***Keyboard***
Letter recognition	0.32*	0.11	0.22	0.55**	0.25	0.08
Letter writing	0.80**	0.81**	0.81**	0.31*	0.32*	0.37*
Word reading	0.70**	0.63**	0.77**	0.38**	0.16	0.21
Word writing	0.71**	0.69**	0.70**	0.33*	0.09	0.36*

Note. T1 = assessment before training; * indicate a significant correlation with $p < 0.05$; ** indicate a significant correlation with $p < 0.01$.

Supplementary Table 3.

Descriptive data of all dependent variables for the pencil group at the different time points of assessment

**Pencil group**	**T1**<br>Mean (*SD*)<br>[Min – Max]	**T2**<br>Mean (*SD*)<br>[Min – Max]	**T3**<br>Mean (*SD*)<br>[Min – Max]
Letter recognition (raw score)	7.00 (2.70) [1 – 11]	8.58 (2.02) [3 – 11]	8.53 (2.05) [4 – 11]
Letter writing (raw score)	6.56 (4.50) [0 – 16]	9.90 (4.17) [1 – 16]	10.21 (4.17) [2 – 16]
Word reading (raw score)	--[1]	1.75 (1.80) [0 – 5]	1.76 (2.02) [0 – 5]
Word writing (raw score in %)	--[1]	49.27 (33.00) [0 – 100]	52.00 (36.00) [4 – 100]
Visuo-spatial skills (raw score)	15.27 (5.57) [5 – 25]	17.35 (5.02) [6 – 24]	--[2]

Note. . T1 = assessment before training; T2 = assessment after training; T3 = assessment 4-5 weeks after training; --[1]: variable was not quantified at T1; --[2]: variable was not quantified at T3.

Supplementary Table 4.

Descriptive data of all dependent variables for the stylus group at the different time points of assessment

**Stylus group**	**T1**<br>Mean (*SD*)<br>[Min – Max]	**T2**<br>Mean (*SD*)<br>[Min – Max]	**T3**<br>Mean (*SD*)<br>[Min – Max]
Letter recognition (raw score)	7.13 (2.19)<br>[1 – 10]	8.51 (2.04)<br>[3 – 11]	8.41 (2.14)<br>[4 – 11]
Letter writing (raw score)	6.20 (3.06)<br>[0 – 12]	9.77 (3.68)<br>[3 – 15]	9.93 (3.90)<br>[1 – 16]
Word reading (raw score)	--[1]	1.17 (1.56)<br>[0 – 5]	1.21 (1.50)<br>[0 – 5]
Word writing (raw score in %)	--[1]	41.94 (28.71)<br>[0 – 100]	48.23 (30.14)<br>[0 – 100]
Visuo-spatial skills (raw score)	15.96 (5.05)<br>[5 – 23]	17.57 (4.12)<br>[5 – 25]	--[2]

Note. . T1 = assessment before training; T2 = assessment after training; T3 = assessment 4-5 weeks after training; --[1]: variable was not quantified at T1; --[2]: variable was not quantified at T3.

Supplementary Table 5.

Descriptive data of all dependent variables for the keyboard group at the different time points of assessment

**Keyboard group**	**T1**<br>Mean (*SD*)<br>[Min – Max]	**T2**<br>Mean (*SD*)<br>[Min – Max]	**T3**<br>Mean (*SD*)<br>[Min – Max]
Letter recognition (raw score)	7.13 (2.02)<br>[2 – 11]	8.00 (2.22)<br>[3 – 11]	7.48 (2.01)<br>[2 – 11]
Letter writing (raw score)	7.74 (4.49)<br>[0 – 15]	11.76 (4.19)<br>[2 – 16]	11.27 (4.32)<br>[1 – 16]
Word reading (raw score)	--[1]	1.98 (1.85)<br>[0 – 5]	2.26 (1.88)<br>[0 – 5]
Word writing (raw score in %)	--[1]	56.78 (34.66)<br>[0 – 100]	58.83 (34.16)<br>[0 – 100]
Visuo-spatial skills (raw score)	16.11 (4.76)<br>[5 – 24]	16.15 (4.57)<br>[4 – 22]	--[2]

Note. . T1 = assessment before training; T2 = assessment after training; T3 = assessment 4-5 weeks after training; --[1]: variable was not quantified at T1; --[2]: variable was not quantified at T3.

Supplementary Table 6.

Results of estimated LMMs for the variable letter recognition (T1 vs. T2)

	**Estimate (*SE*)**	***df***	***t*-Score**	***p*-Value**	***CI* 95%**	
					**Lower**	**Upper**
Reference: pencil group						
Intercept	7.06 (.30)	274.26	23.27	<.001	6.46	7.65
T3 (Reference=T1)	1.56 (.41)	141.60	3.78	<.001	.74	2.37
Stylus group	.10 (.43)	274.11	.23	.821	-.75	.94
Keyboard group	-.02 (.43)	274.13	-.04	.968	-.87	.83
Visuo-spatial skills (at T1)	.06 (.03)	139.35	2.20	.029	.01	.11
Letter reading (at T1)	.11 (.03)	139.33	4.11	<.001	.06	.16
T3 * stylus group	-.20 (.58)	140.87	-.34	.738	-1.35	.96
T3 * keyboard group	-.71 (.59)	140.88	-1.21	.230	-1.87	.45
Reference: keyboard group						
Intercept	7.04 (.31)	274.08	22.95	<.001	6.43	7.64
T3 (Reference=T1)	. 58 (.42)	140.18	2.03	.044	.02	1.68
Stylus group	.11 (.43)	274.02	.27	.791	-.74	.96
Pencil group	-.02 (.43)	274.13	.04	.968	-.83	.87
Visuo-spatial skills (at T1)	.06 (.03)	139.35	2.20	.029	.01	.11
Letter reading (at T1)	.11 (.03)	139.33	4.11	<.001	.06	.16
T3 * stylus group	.51 (.59)	140.17	.87	.386	-.65	1.68
T3 * pencil group	.71 (.59)	140.88	1.21	.230	-.45	1.87

Supplementary Table 7.

Results of estimated LMMs for the variable letter recognition (T2 vs T3)

	**Estimate (*SE*)**	***df***	***t*-Score**	***p*-Value**	***CI* 95%**	
					**Lower**	**Upper**
Reference: pencil group						
Intercept	8.63 (0.29)	224.57	29.97	<.001	8.07	9.20
T3 (Reference=T2)	-0.05 (.30)	133.67	-.17	.867	-.64	.54
Stylus group	-0.16 (.41)	222.89	-.40	.693	-.97	.65
Keyboard group	-0.72 (.41)	224.50	-1.74	.083	-1.53	.09
Visuo-spatial skills (at T1)	.10 (.03)	140.00	3.15	.002	.04	.16
Letter reading (at T1)	.05 (.03)	139.21	1.56	.121	-.01	.11
T3 * stylus group	-.30 (.56)	134.88	-.26	.799	-.96	.74
T3 * keyboard group	-1.15 (.56)	135.59	-1.00	.319	-1.28	.42
Reference: keyboard group						
Intercept	7.92 (.29)	224.83	26.85	<.001	7.34	8.50
T3 (Reference=T2)	-.48 (.31)	137.34	-1.55	.125	-1.10	.13
Stylus group	.56 (.42)	223.31	1.34	.182	-.26	1.38
Pencil group	.72 (.41)	224.50	1.74	.083	-.09	1.53
Visuo-spatial skills (at T1)	.10 (.03)	140.00	3.15	.002	.04	.16
Letter reading (at T1)	.05 (.03)	139.21	1.56	.121	-.01	.11
T3 * stylus group	.32 (.44)	136.70.	.73	.464	-.55	1.19
T3 * pencil group	.43 (.43)	135.59	1.00	.319	-.42	1.28

Supplementary Table 8.

Results of estimated LMMs for the variable letter writing (T1 vs. T2)

	**Estimate (*SE*)**	***df***	***t*-Score**	***p*-Value**	***CI* 95%**	
					**Lower**	**Upper**
Reference: pencil group						
Intercept	6.90 (.32)	257.47	21.45	<.001	6.27	7.53
T2 (Reference=T1)	3.37 (.39)	139.95	8.69	<.001	2.61	4.14
Stylus group	-.10 (.45)	256.61	-.22	.828	-.99	.79
Keyboard group	.37 (.46)	257.44	.81	.420	-.54	1.28
Visuo-spatial skills (at T1)	.04 (.03)	139.45	1.42	.157	-.02	.10
Letter reading (at T1)	.66 (.03)	139.12	21.20	<.001	.59	.72
T2 * stylus group	.30 (.55)	139.94	.55	.584	-.78	1.39
T2 * keyboard group	.62 (.56)	140.01	1.12	.263	-.47	1.72
Reference: keyboard group						
Intercept	7.27 (.33)	257.45	22.11	<.001	6.62	7.92
T2 (Reference=T1)	4.00 (.40)	140.07	10.07	<.001	3.21	4.78
Stylus group	.47 (.46)	256.53	-1.03	.306	-1.37	.43
Pencil group	-.37 (.46)	257.44	-.81	.420	-1.28	.54
Visuo-spatial skills (at T1)	.04 (.03)	139.45	1.42	.157	-.02	.105
Letter reading (at T1)	.66 (.03)	139.12	21.20	<.001	.59	.72
T2 * stylus group	-.32 (.56)	139.99	-.58	.563	-1.42	.78
T2 * pencil group	-.62 (.56)	140.01	-1.12	.263	-1.72	.475

Supplementary Table 9.

Results of estimated LMMs for the variable letter writing (T2 vs. T3)

	**Estimate (*SE*)**	***df***	***t*-Score**	***p*-Value**	***CI* 95%**	
					**Lower**	**Upper**
Reference: pencil group						
Intercept	10.25 (.37)	169.53	27.56	<.001	9.52	10.99
T3 (Reference=T2)	.16 (.24)	129.85	.63	.528	-.33	.64
Stylus group	.10 (.53)	169.53	.19	.852	-.95	1.14
Keyboard group	1.13 (.53)	169.48	2.12	.035	.08	2.18
Visuo-spatial skills (at T1)	.09 (.04)	139.35	2.22	.028	.01	.18
Letter reading (at T1)	.60 (.04)	137.80	14.04	<.001	.51	.68
T3 * stylus group	-.09 (.35)	130.73	-.24	.808	-.79	.61
T3 * keyboard group	-.47 (.35)	130.46	-1.33	.185	-1.17	.23
Reference: keyboard group						
Intercept	11.38 (.38)	169.68	29.93	<.001	10.63	12.13
T3 (Reference=T2)	-.32 (.26)	131.02	-1.24	.217	-.82	.19
Stylus group	-1.03 (.44)	169.65	-1.93	.056	-2.09	.03
Pencil group	-1.13 (.44)	169.48	-2.12	.035	-2.18	-.08
Visuo-spatial skills (at T1)	.09 (.04)	139.35	2.22	.028	.010	.18
Letter reading (at T1)	.60 (.04)	137.80	14.04	<.001	.51	.68
T3 * stylus group	.39 (.36)	131.29	1.07	.287	-.33	1.10
T3 * pencil group	.47 (.35)	130.46	1.33	.185	-.23	1.17

Supplementary Table 10.

Results of estimated LMMs for the variable letter writing (T1 vs. T3)

	**Estimate (*SE*)**	***df***	***t*-Score**	***p*-Value**	***CI* 95%**	
					**Lower**	**Upper**
Reference: pencil group						
Intercept	6.90 (.33)	245.63	20.61	<.001	6.24	7.56
T3 (Reference=T1)	3.55 (.39)	137.12	9.19	<.001	2.78	4.31
Stylus group	-.10 (.47)	244.55	-.21	.838	-1.03	.83
Keyboard group	.38 (.48)	245.64	.80	.425	-.56	1.33
Visuo-spatial skills (at T1)	.05 (.03)	147.03	1.51	.133	-.02	.12
Letter reading (at T1)	.65 (.03)	141.84	19.68	<.001	.59	.72
T3 * stylus group	.26 (.55)	139.10	.48	.632	-.83	1.36
T3 * keyboard group	.18 (.56)	138.60	.33	.745	-.92	1.28
Reference: keyboard group						
Intercept	7.28 (.34)	245.63	21.27	<.001	6.60	7.95
T3 (Reference=T1)	3.73 (.40)	140.05	9.31	<.001	2.94	4.52
Stylus group	-.48 (.48)	244.47	-1.00	.316	-1.42	.46
Pencil group	-.38 (.48)	245.64	-.80	.425	-1.33	.56
Visuo-spatial skills (at T1)	.05 (.03)	147.03	1.51	.133	-.02	.12
Letter reading (at T1)	.65 (.03)	141.84	19.68	<.001	.59	.72
T3 * stylus group	.08 (.56)	140.46	.15	.882	-1.03	1.20
T3 * pencil group	-.18 (.56)	138.60	-.33	.745	-1.28	.92

Supplementary Table 11.

Results of estimated LMMs for the variable word writing (T2 vs. T3)

	**Estimate (*SE*)**	***df***	***t*-Score**	***p*-Value**	***CI* 95%**	
					**Lower**	**Upper**
Reference: pencil group						
Intercept	49.29 (3.43)	163.77	14.37	<.001	42.51	56.06
T3 (Reference=T2)	3.42 (1.96)	132.85	1.74	.084	-.46	7.31
Stylus group	-5.62 (4.88)	162.89	-1.15	.251	-15.25	4.01
Keyboard group	3.75 (4.91)	163.79	.76	.446	-5.94	13.44
Visuo-spatial skills (at T1)	.37 (.40)	141.07	.92	.360	-.42	1.16
Letter reading (at T1)	4.33 (.40)	139.39	10.89	<.001	3.54	5.11
T3 * stylus group	2.65 (2.84)	133.49	.93	.352	-2.96	8.26
T3 * keyboard group	1.38 (2.85)	133.59	.48	.629	-4.26	7.03
Reference: keyboard group						
Intercept	53.04 (3.51)	163.89	15.13	<.001	46.11	59.96
T3 (Reference=T2)	4.80 (2.07)	134.24	2.32	.022	.71	8.90
Stylus group	-9.37 (4.93)	162.92	-1.90	.059	-19.11	.37
Pencil group	-3.75 (4.91)	163.79	-.76	.446	-13.44	5.94
Visuo-spatial skills (at T1)	.37 (.40)	141.07	.92	.360	-.42	1.16
Letter reading (at T1)	4.33 (.40)	139.39	10.90	<.001	3.54	5.11
T3 * stylus group	1.27 (2.91)	134.15	.44	.664	-4.49	7.03
T3 * pencil group	-1.38 (2.85)	133.59	-.48	.629	-7.03	4.26

Supplementary Table 12.

Results of estimated LMMs for the variable word reading (T2 vs. T3)

	**Estimate (*SE*)**	***df***	***t*-Score**	***p*-Value**	***CI* 95%**	
					**Lower**	**Upper**
Reference: pencil group						
Intercept	1.78 (.18)	183.70	9.62	<.001	1.41	2.14
T3 (Reference=T1)	-.003 (.14)	131.26	-.02	.981	-.30	.28
Stylus group	-.47 (.26)	182.39	-1.81	.072	-.99	.04
Keyboard group	-.02 (.26)	181.95	-.07	.948	-.54	.50
Visuo-spatial skills (at T1)	.01 (.02)	139.76	.59	.559	-.03	.05
Letter reading (at T1)	.30 (.03)	137.84	11.72	<.001	.25	.35
T3 * stylus group	.06 (.21)	132.33	.31	.760	-.34	.47
T3 * keyboard group	.36 (.21)	132.46	1.74	.085	-.05	.77
Reference: keyboard group						
Intercept	1.76 (.19)	180.90	9.34	<.001	1.39	2.13
T3 (Reference=T1)	.36 (.15)	133.55	2.37	.019	.06	.66
Stylus group	-.46 (.26)	180.75	-1.72	.086	-.97	.07
Pencil group	.02 (.26)	181.95	.07	.948	-.50	.54
Visuo-spatial skills (at T1)	.01 (.02)	139.76	.59	.559	-.03	.05
Letter reading (at T1)	.30 (.03)	137.84	11.72	<.001	.25	.35
T3 * stylus group	-.30 (.21)	133.47	-1.41	.160	-.72	.12
T3 * pencil group	-.36 (.21)	132.46	-1.74	.085	-.77	.05
